# Structure and Dynamics of an Archeal Monoglyceride Lipase from *Palaeococcus ferrophilus* as Revealed by Crystallography and In Silico Analysis

**DOI:** 10.3390/biom11040533

**Published:** 2021-04-03

**Authors:** Geoffray Labar, Nathalie Brandt, Amaury Flaba, Johan Wouters, Laurence Leherte

**Affiliations:** 1 Institut de Recherches Labiris, 1 Avenue E. Gryson, B-1070 Bruxelles, Belgium; nbrandt@spfb.brussels (N.B.); amaury_flaba@yahoo.fr (A.F.); 2 Laboratoire de Chimie Biologique Structurale, Department of Chemistry, Namur Medicine & Drug Innovation Center (NAMEDIC-NARILIS), Namur Research Institute for Life Sciences (NARILIS), University of Namur, Rue de Bruxelles 61, B-5000 Namur, Belgium; johan.wouters@unamur.be; 3 Laboratoire de Chimie Biologique Structurale, Department of Chemistry, Namur Medicine & Drug Innovation Center (NAMEDIC-NARILIS), Namur Institute of Structured Matter (NISM), University of Namur, Rue de Bruxelles 61, B-5000 Namur, Belgium

**Keywords:** monoglyceride lipase, monoacylglycerol lipase, crystallography, molecular dynamics simulation, elastic network model

## Abstract

The crystallographic analysis of a lipase from *Palaeococcus ferrophilus* (PFL) previously annotated as a lysophospholipase revealed high structural conservation with other monoglyceride lipases, in particular in the lid domain and substrate binding pockets. In agreement with this observation, PFL was shown to be active on various monoacylglycerols. Molecular Dynamics (MD) studies performed in the absence and in the presence of ligands further allowed characterization of the dynamics of this system and led to a systematic closure of the lid compared to the crystal structure. However, the presence of ligands in the acyl-binding pocket stabilizes intermediate conformations compared to the crystal and totally closed structures. Several lid-stabilizing or closure elements were highlighted, i.e., hydrogen bonds between Ser117 and Ile204 or Asn142 and its facing amino acid lid residues, as well as Phe123. Thus, based on this complementary crystallographic and MD approach, we suggest that the crystal structure reported herein represents an open conformation, at least partially, of the PFL, which is likely stabilized by the ligand, and it brings to light several key structural features prone to participate in the closure of the lid.

## 1. Introduction

Lipases and esterases are of great value as biocatalysists in diverse industrial applications. Esterification, transesterification, or hydrolysis by lipases allow the synthesis or degradation of a wide range of compounds of interest in the pharmaceutical, food, textile, or paper industries, as well as in environmental applications for the synthesis and recycling of biodegradable polymers [1,2,3,4]. They are also in use as biocatalysts for the production of biodiesels, which are an important renewable fuel source [2,4,5]. The regio- and stereoselectivity of lipases as well as their compatibility with organic solvents and thermostability are among the key strengths offered by lipases.

In addition to their use in the industry, lipases are also of great biological importance in all domains of life. Among the lipases, monoglyceride lipases (MGLs) hydrolyze fatty acid monoesters (EC 3.1.1.23) and are of particular interest, since they have been shown to be involved in the energy homeostasis or in signaling functions involving lipids. For instance, since the enzyme was shown to constitute a key actor of the endocannabinoid signaling system, a great deal of attention was devoted to the human monoglyceride lipase (hMGL), which is now considered as a promising drug target for the treatment of several disorders including cancer and inflammatory diseases [6,7]. Homologs of hMGL were also identified in bacteria, yeast, and recently, mycobacterium tuberculosis MGL (mtMGL) inhibition emerged as a new therapeutic strategy to treat tuberculosis [8].

Whether it be in the industrial or biological context, an accurate understanding of the functioning of lipases is expected. In the industry, a careful selection of the best lipase—or its engineering—is required to meet the criteria for each particular purpose. In the biological or therapeutical perspective, an in-depth molecular comprehension of the functioning of lipases in general is also highly desired.

With this in mind, during our study of the hMGL, a search for homologs led us to the identification of two lipases from *Palaeococcus ferrophilus* (PFL) and *Thermococcus barophilus* (TBL). The enzymes, which were classified as lysophospholipases, drew our attention due to the relatively high homology found in the cap subdomain compared to other MGLs described to date. Many lipases have evolved to contain a so-called cap domain, which, in MGLs, consists in an insertion after the β6 sheet of the α/β hydrolase core domain and participates in the substrate recognition or serves as a lid to control the access to the active site [9]. The primary sequence in this region is often highly divergent throughout lipases, even with similar function. Nevertheless, the three-dimensional architecture of the lid was recently suggested to constitute a hallmark of α/β hydrolase with MGL activity [10]. PFL and TBL share with hMGL 39% identity in the cap region, which is a higher score than that calculated for the whole amino acid (AA) sequence (32 and 33%) [11]. The homology between the cap subdomain of the archaeal lipases and hMGL is also the highest compared to that of all other MGLs of known structure (Figure S1).

PFL, as well as TBL, were first annotated as lysophospolipases in databases [12]. In the ESTHER classification (ESTerases, alpha/beta-Hydrolase Enzymes and Relatives), PFL and MGL belong to the MGL/lysophospholipase family in the hydrolase four-parent family of the X block. In the LED database, α/β hydrolases are also classified as GX- or GGGX-type. According to this classification, PFL oxyanion is of the GX-type, meaning that the first oxyanion hole residue is preceded by one glycine residue [13].

In this work, the three-dimensional structure of PFL was determined. The structure was compared to other α/β hydrolases, which, based on the structural homology with other MGLs, led us to reassign a MGL activity to the enzyme.

Elastic Network Model analysis (ENM) and Molecular Dynamics (MD) simulations were also carried out to characterize the PFL dynamics and to evaluate whether its crystal structure adopts an open or closed conformation. The lid domain movements have already been studied through MD simulations, at 300 K, for periods of time ranging from 20 to 100 ns. Particularly, Pleiss and co-workers studied the conformational transitions of several lipase systems as a function of the solvent [14,15]. They found out that closed conformations are stable in water, while the opening is observed in toluene. In the case of the hMGL, Chen et al. [16] identified that the presence (or the absence) of the substrate affects the enzyme conformation. The authors especially focused on the distance between Phe159 of the helix H4 of the lid and Gly210 of H6 to differentiate the closed and open conformations. Riccardi et al. studied hMGL embedded in a lipid bilayer through longer 500 ns MD simulations with different substrates [17]. In addition to Phe159, which is depicted as an interacting residue for a substrate with unsaturated bonds, the authors identified Ile179 as a “gatekeeper” that regulates the ligand entrance. In a recent work, Ali et al. carried out MD simulations of a diacylglycerol lipase and analyzed the role of particular residues, Phe278 and Gln282, in the substrate specificity [18].

The present studies of the enzyme in interaction with lauryl dimethylamine oxide (LDAO) or the covalently bound methyl arachidonyl fluorophosphonate (MAFP), and glycerol, were achieved to highlight structural elements that favor the conformational states.

## 2. Materials and Methods

### 2.1. Experimental Methods

#### 2.1.1. Expression and Purification of PFL

The gene encoding PFL was synthetically generated (GeneArt, Thermo Fisher Scientific, Waltham, MA, USA) and cloned in the pET30b plasmid as a NdeI/XhoI fragment. Then, the *E.coli* BL21(DE3) strain was transformed with the expression vector using standard procedures and grown in rich medium at 37 °C until an optical density of 0.6 was reached. Then, isopropyl-β-d-thiogalactopyranoside 0.3 mM was added, and the culture was maintained at 20 °C for 18 h. Bacteria were harvested by centrifugation, resuspended in buffer A (KH_2_PO_4_/K_2_HPO_4_ 100 mM, imidazole 10 mM, NaCl 150 mM, LDAO 0.5%, pH 8.1), supplemented with Dnase 2.5 µg/mL, and lysed by sonication for 20 min at 4 °C. After a centrifugation (20,000× *g*, 30 min, 4 °C) to clear the lysate, the supernatant was submitted to a heat shock at 65 °C during 10 min. Insoluble proteins were removed by another purification step (20,000× *g*, 30 min, 4 °C), and the sample was submitted to further purification. Briefly, the sample was loaded on a Chelating Sepharose Fast Flow resin loaded with Ni^++^ ions. After washing the column with buffer A, an imidazole gradient using buffer A supplemented with 400 mM imidazole was applied to elute the protein of interest. Pooled fractions corresponding to PFL were concentrated by ultrafiltration (Amicon^®^, 10 kDa cut-off, MilliporeSigma, Burlington, MA, USA) and further purified by gel filtration chromatography, using a Superose 12 10/300 GL column in buffer C (Hepes 20 mM, NaCl 150 mM, pH 8.1) supplemented with LDAO 0.1%, unless otherwise indicated. The fractions of interest were concentrated again until a concentration of 15 mg/mL was reached. The purity of the protein was assessed by SDS-PAGE with Coomassie blue staining and shown to be above 95%. Glycerol 10% (*v/v*) was added to the sample, which was frozen in liquid nitrogen and stored at −80 °C until further analysis.

#### 2.1.2. Crystallization, Data Collection, and Structural Characterization of PFL

Crystallogenesis was performed by the under oil crystallization procedure. First, 1 µL of pure PFL (15 mg/mL) was mixed with 1 µL of crystallization solution, and the drop was covered with 18 µL of paraffin oil and incubated at 20 °C. The best crystals were obtained with solution #10 of the JCSGplus suite from Molecular Dimensions (PEG3350 20% (*m*/*v*), potassium formate 0.2 M). No further improvement was obtained during the optimization procedure. Crystals, which grew in 2–7 days, were analyzed at the SOLEIL Proxima 2 beamline, France. The XDS [19] and Phenix packages [20] were used for the processing of collected data and model building and refinement, respectively. The structure was solved by molecular replacement using hMGL (pdb code 3hju [7]). Data collection and refinement statistics are presented in Table S1. PyMOL was used as a molecular graphic tool and to prepare the figures [21]. The PDBSum software was used to create a 2D topology diagram of PFL (Figure S2) [22]. A feature-enhanced map was generated, as well as a Polder map, to highlight the presence of LDAO (Figure S3) [23,24].

#### 2.1.3. Thin Layer Chromatography Lipase Assay

PFL lipase activity against lysophosphatidylcholine (LPC), lysophosphatidylethanolamine (LPE), and various monoacylglycerols was assayed by incubating 0.5 µg PFL with 12.5 µg of lysophosphatidylcholine (Sigma-Aldrich, St Louis, MI, USA, 0.5 mM final concentration), lysophosphatidylethanolamine (Sigma-Aldrich), decanoyl glycerol (DG, Sigma-Aldrich, 1 mM final concentration), myristoyl glycerol (MG, Sigma-Aldrich, 0.8 mM final concentration) or oleoyl glycerol (OG, Sigma-Aldrich, 0.7 mM final concentration) in Hepes Buffer (Hepes 100 mM, pH 8) during 20 min at 50 °C, in a total reaction volume of 50 µL. At the end of the incubation, 50 µL of chloroform/methanol/acetic acid in a volumetric ratio of 50:50:1 (*v/v/v*) were added, and the sample was centrifugated for 1 min at 1000× *g*. Then, 8 µL of the organic phase were spotted on a silica gel 60W TLC plate, and the lipids were separated using a mixture of hexane-diethylether 60:40 (*v/v*) as eluent. Primuline, as a 0.5 mg/mL solution in an acetone–water 80:20 (*v/v*) mixture, was sprayed on the plate to stain the lipids, which were revealed by fluorescence on a Chemi Premium imager (blue illumination, 525 nm emission filter). Myristic and oleic acids were used as standards for the identification of the hydrolysis product.

#### 2.1.4. pNitrophenyl Esters Hydrolase Assay

The hydrolase activity was also assessed using p-nitrophenyl esters of various acyl chain lengths through the measure of the colorimetric p-nitrophenol produced upon hydrolysis. The protein (675–1350 ng), diluted in a total volume of 120 µL reaction buffer (Hepes 100 mM, pH 8, supplemented or not with LDAO 0.1%), was mixed with 80 µL of the substrates dissolved in DMSO to reach a final concentration of 1 mM (4-Nitrophenyl acetate, 4-Nitrophenyl butyrate, 4-Nitrophenyl octanoate, 4-Nitrophenyl decanoate, 4-Nitrophenyl dodecanoate, 4-Nitrophenyl myristate, 4-Nitrophenyl palmitate; Sigma-Aldrich, St Louis, MI, USA). The reaction was allowed to proceed at either 30 or 70 °C, and the activity was detected by measuring the absorbance at 445 nm using a Biotek PowerWave XS2 spectrophotometer, after stopping the reaction by dipping the tubes in ice-cold water and adding 7.5 µL of methyl arachidonyl fluorophosphonate (MAFP, Sigma-Aldrich, St Louis, MI, USA) 2 × 10^−5^ M. The amount of generated *p*-nitrophenol was calculated based on a calibration curve, after the subtraction of blank values. When measuring the MAFP inhibitory potency, 10 µL of the inhibitor at 20 times working concentration were added to the reaction mixture. GraphPad Prism software was in this case used to determine the pIC50. At least three independent experiments were performed for each reaction.

#### 2.1.5. Thermofluor Assay

The stability of PFL was assessed using the thermofluor assay. Then, 2 µL of a 50 µM PFL solution were mixed with 1 µL MAFP 1 mM or DMSO and 14.5 µL of reaction buffer (Hepes 100 mM, pH 8, supplemented or not with LDAO 0.1%). Then, 2.5 µL of a 40× concentrated SYPRO^TM^ Orange solution (Thermo Fisher Scientific, Waltham, MA, USA), prepared by a 125 fold dilution of a proprietary 5000 fold concentrate solution in the reaction buffer, were added, and the samples were analyzed on a StepOnePlus Real Time PCR system. A temperature gradient from 15 to 98 °C was applied, with a 1% slope (internal units). The StepOnePlus Software was used to analyze the fluorescence data and to derive the melting temperature (Tm), which was defined as the minimum of (-) the first derivative of the fluorescence versus temperature plot (‘–dF/dT’ plot). For each condition, the value is the mean of at least four independent experiments.

### 2.2. Computational Investigations

**Figure 1:**
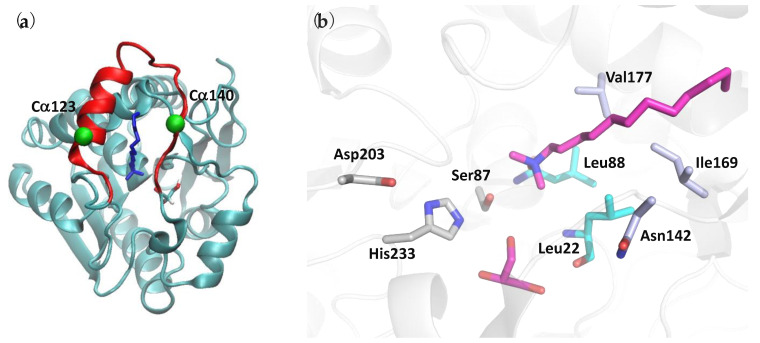
(**a**) Crystal structure of the chain A of *Palaeococcus ferrophilus* (PFL) (cyan), with lauryl dimethylamine oxide (LDAO) and glycerol molecules (sticks), and Cα123 and Cα140 (green spheres). The sequence 120 to 144, which is comprised in the cap, is in red. (**b**) View of the PFL active site, with key residues and a co-crystallized LDAO molecule represented as sticks. Carbon atoms are colored as followed: gray, catalytic triad; cyan, oxyanion hole; light blue, Leu22 stabilizing residues; magenta, LDAO and glycerol.

To model the enzyme, the chain A of the PFL crystal structure was selected. The main conformation was retained for residues with a double conformation, i.e., Lys80, Ser141, Asp179, Met181, and Ser255. The enzyme contains 257 amino acid residues, and several helices characterize its secondary structure (Table S2). According to Riegler-Berket, the PFL cap involves residues 115 to 169 [10] and comprises the helix H5 which, in hMGL, was shown to constitute the mobile lid of the enzyme, and its facing loop (Figure 1) [7,25,26]. The LDAO and glycerol conformations were taken from the crystal structure.

#### 2.2.1. Elastic Network Models

ENM consists in calculating the 3N-6 normal *mode* of vibration of a protein structure described using a limited set of masses. Usually, each AA residue is represented by one grain, which is placed at the Cα. Atoms interact with each other depending upon their separation distance through an interaction potential which, in its simplest form, mimics a harmonic spring. The Hessian matrix is diagonalized so that eigenvalues (vibration frequencies) and eigenvectors (displacement vectors) are obtained. Calculations were carried out with the server iMODS [27]. To solve the motion equation, the edNMA model was selected [28]. It is based on Cα–Cα force constants that were determined from MD trajectories of various protein systems and does not require any user-specified cut-off values.

#### 2.2.2. Molecular Dynamics Simulations

In the present work, MD simulations were carried out using the program Gromacs5.1.4 [29]. The chain A of PFL was modeled under various simulation conditions, i.e., either uncomplexed, at two different temperatures, or interacting with ligands. The united-atom Gromos54a7 force field (FF) [30] was used to model the protein (PFL), glycerol, and the ligands, such as LDAO and methyl arachidonyl fluorophosphonate (MAFP). Glycerol and LDAO coordinates were taken from the crystal structure (Figure 1). The structure of the last ligand that covalently binds to the protein receptor is taken from the PDB structure file 1mt5. It is covalently linked to Ser87 of PFL. The ligand preparation is described in the Supplementary Material SM1. The FF parameters are available for the small molecules through the automated topology builder ATB [31]. All systems were solvated in a box of Simple Point Charge (SPC) water molecules as recommended with the program Gromacs5.1.4 [29], and one chloride ion is added to neutralize the total charge of the systems. The box size is selected so as to achieve a distance of at least 1.2 nm between the protein surface and the box limits. The systems under study are described in Table 1. They are first optimized and then progressively heated up to 300 K, and the final MD production stage is carried out for a period of 300 ns (Supplementary Material SM2). As reminded later, the analyses were eventually carried out on the last 200 ns of the production stage. The VMD program was used to visualize the MD snapshots [32].

## 3. Results

### 3.1. Experimental Results

#### 3.1.1. PFL Overall Structure

PFL was expressed in *E. coli* and purified by affinity chromatography and gel filtration. The structure of the enzyme, which crystallized in space group C2, was solved by molecular replacement, using hMGL as a template. The PFL overall structure is composed of an α/β hydrolase core, consisting of a central β-sheet surrounded by six α-helices (Figure 1 and Figure S2) [22]. Only seven β-strands constitute the central β-sheet. This deviation from the canonical α/β hydrolase fold, which originates from the lack of the first β-hairpin at the beginning of the β-sheet, is similar to what was observed for *Bacillus sp.* H257 MGL (bMGL) for instance [33].

Two molecules are present in the asymmetric unit, which is in line with the dimeric form observed by size exclusion chromatography. An LDAO molecule is present in the active site (Figure 1a). Although we cannot exclude that other buffer molecules fill the electron density instead of LDAO, the calculated maps, including “feature-enhanced modification” [23] and Polder maps (Figure S3) [24], together with the fact that the absence of LDAO impedes crystallization, are in good agreement with the presence of the detergent in the active site. Interestingly, removing the detergent from the buffer also yields to a higher molecular size protein (4-mer to 7-mer).

The catalytic triad is made of Ser87, His233, and Asp203 (Figure 1b). The PFL oxyanion hole, which stabilizes the transition state intermediate, is composed of the NH groups of Leu 88 and Leu 22. The side chain of Leu22 is stabilized by a hydrophobic patch formed by the side chain of Leu173, Ile169, Leu88, and Asn142 anchor residues, which are located at approximately a 3.5–4.3 Å distance.

#### 3.1.2. Homology with hMGL and mtMGL

- *Hinge and cap conformation.* A DALI search retrieved hMGL as the first hit [7,25,26,34]. Other close structural homologs of PFL include mtMGL and *Saccharomyces cerevisiae* (scMGL) monoglyceride lipases [8,35]. Despite the relatively low sequence identity with these lipases (32%, 37%, and 23% identity with hMGL, mtMGL, and scMGL, respectively) the enzymes retain a striking conservation of structural elements, which are presumed to be of critical importance to support the activity (Figure S1).

**Figure 2:**
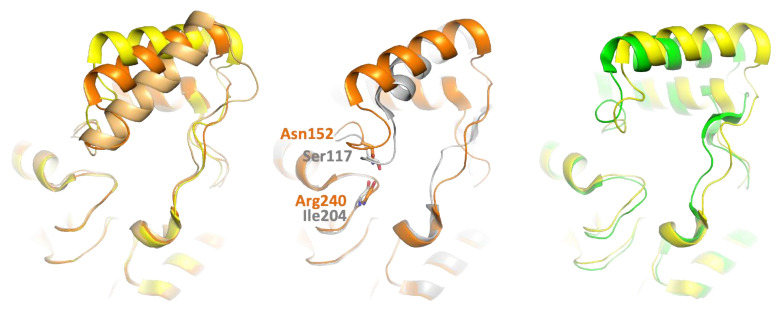
Comparison of the structures of PFL (gray), mtMGL (pdb code 6eic, chain B, green), semi-open hMGL (pdb code 3jw8, chain A, dark orange), fully open hMGL (pdb code 4uuq, chain B, yellow), and closed hMGL (pdb code 3pe6, light orange). PFL cap, and in particular the conformation of the *N*-term hinge of the H5 helix, is closer to that of semi-open MGL, whilst mtMGL resembles more the fully open form of hMGL (see also in the text). A hydrogen bond between the Ile204 main chain and the Ser117 side chain stabilizes the *N*-term hinge of H5 (represented as sticks). A similar interaction is observed in semi-open hMGL but is absent in the fully open hMGL and mtMGL.

Whilst the cap region (i.e., residues 115–169 in PFL) is the most variable region, a very good overlay can be obtained, which shows that the cap organization is remarkably well conserved between PFL, hMGL, and mtMGL (Figure S4). In the three enzymes, the presumed lid comprises the amphipathic H5 α-helix. Residues 135-144 connect H5 to H6, and this region is named linker H56 further in the text. Several structures of hMGL with alternate lid conformations have been observed experimentally [7,9,17,25,26,35,36,37]. In addition to the fully open and closed states, the enzyme was captured in another open conformation, which, considering the *N*-terminal hinge of H5, can be viewed as an intermediate state between these extreme conformations and is hereafter named semi-open hMGL. Actually, whilst mtMGL is presumed to have a lid that superposes very well with open hMGL, for PFL, it is closer to the third, semi-open hMGL, especially in terms of the orientation of the H5 helix and the conformation of residues 116-121 in the *N*-terminal hinge (Figure 2). Compared to the closed conformation of the hMGL, the elongation and rotation of H5 in not seen in PFL [26]. In the crystal structure of PFL, Ile204 main chain oxygen is involved in a hydrogen bond with Ser117 Oγ, and it may thus act as a constraint for H5 positioning. Whilst it is absent in the fully open form of hMGL, a similar interaction involves the corresponding residues in the semi-open conformation, i.e., Arg240 carbonyl and Asn152 side chain (the numbering of hMGL residues considered in this work refers to the short isoform of hMGL).

**Figure 3:**
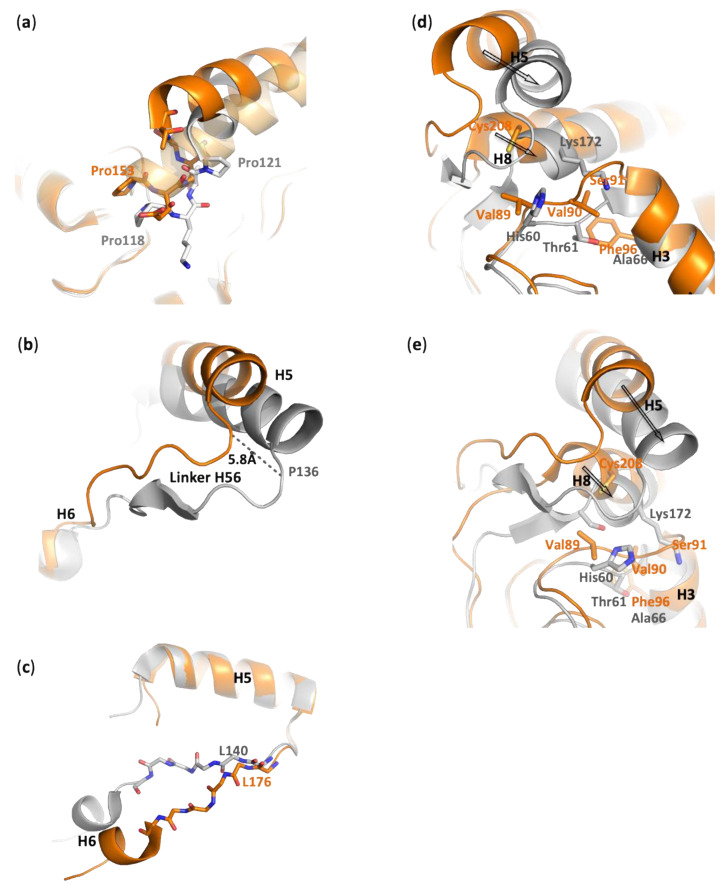
Comparison of *Palaeococcus ferrophilus* (PFL) (gray) and semi-open human monoglyceride lipase (hMGL) (pdb code 3jw8, chain A, orange). (**a**) The *N*-term hinge of PFL is one residue shorter compared to hMGL and contains an additional proline residue, Pro121, which should prevent a similar closure mechanism as seen in hMGL (pdb code 3pe6, light orange). (**b**) The slightly differently oriented H5 helices result in Pro136 residue being located 5.8 Å away from the corresponding hMGL residue. Then, the linker H56 converges at the beginning of H6. (**c**) An alignment of H5 helix highlights the close structural homology between hMGL and PFL, despite their different orientation. The linker H56 diverges slightly from residues 140, because of different backbone torsions angles between residues 140 and 143. (**d**,**e**) Compared to hMGL, modifications in the primary sequence trigger a slight bent of H8 and the concerted motion of the *C*-term hinge and the end of H5.

Despite the fact that H5 helices superpose rather well, both structurally and in terms of sequence conservation, their relative orientation, governed by the hinges and the residues 135–144 between H5 and H6, differs somehow between the PFL, hMGL, and mtMGL. Indeed, the *N*-terminal hinge of hMGL H5 is longer by one residue compared to that of PFL and mtMGL. The constraints imposed by this additional residue in hMGL, as well as the presence of a second proline residue (Pro121) in the PFL hinge compared to hMGL, may contribute to the slightly different positioning of H5 above the active site (Figure 3a). The hinge of mtMGL lacks these two proline residues and is of the same length compared to PFL. However, the psi torsion angle of the main chain backbone of mtMGL differs at residues 138–140 compared to hMGL and PFL and explains the different hinge conformation and lid positioning (Figure S4b).

At the other extremity, the *C*-term end of H5 is shifted by 5.8 Å compared to hMGL (for the sake of clarity, mtMGL is omitted in this section). Then, the path traced by the linker H56 diverges to superimpose again at the level of H6 (Figure 3b). The shorter loop preceding H3 in PFL (residues 89–91) compared to hMGL (residues 60–61) and the introduction of two bulkier residues (i.e., Val89 and Cys208 in hMGL replaced by His60 and Lys172 in PFL) trigger a bent of H8 helix and the concerted motion of the extremity of H5 and the *C*-term hinge (Figure 3d,e). Nevertheless, despite their slightly different orientation, an overlay of the H5 helices of PFL and hMGL highlights their high structural homology. Indeed, a near perfect alignment is obtained in the H5 helix and its *C*-term hinge until residues 140–143, where different backbone torsion angles cause a slight deviation of the main chain path compared to hMGL (Figure 3c).

The PFL lid bears two positively charged residues (Lys129 and Arg133), and its electrostatic potential distribution is only very slightly positive due to the presence of other neighboring arginine (Arg58, Arg168) and lysine (Lys57, Lys172) residues (Figure S5A).

**Figure 4:**
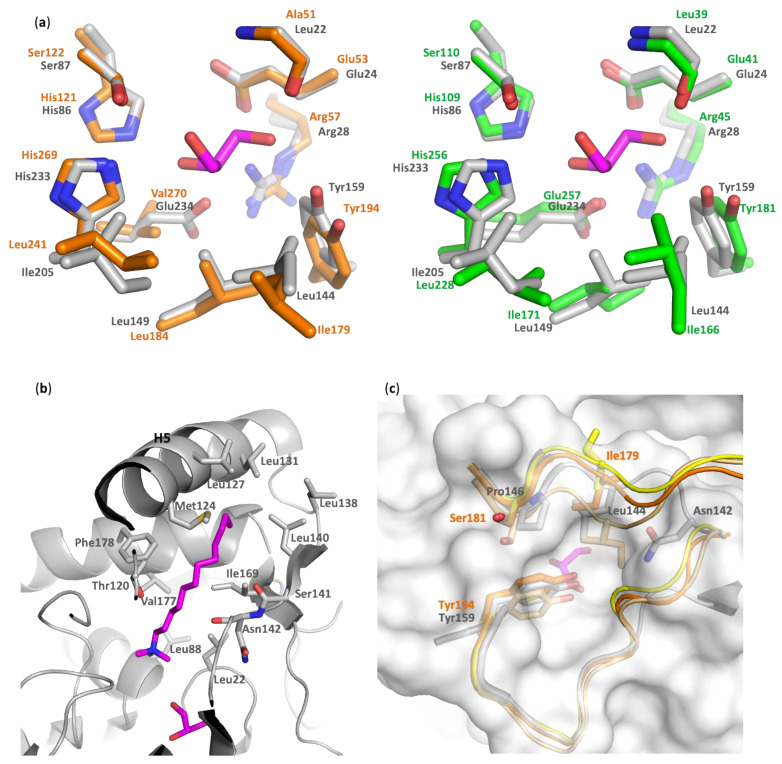
Comparison of the alcohol- and acyl-binding pockets as well as the glycerol exit holes in PFL, hMGL, and mycobacterium tuberculosis MGL (mtMGL). Carbon atoms are colored as follows: gray, PFL; orange, semi-open MGL (pdb code 3jw8, chain A); green, mtMGL; yellow, open MGL (pdb code 4uuq, chain B); light orange, closed hMGL (pdb code 3pe6); magenta, glycerol, and LDAO. (**a**) High conservation of the alcohol-binding pocket of PFL, hMGL, and mtMGL. Key residues and a co-crystallized glycerol molecule are represented as sticks. (**b**) Acyl-binding pocket of PFL. Key residues at the vicinity of the co-crystallized detergent molecule are represented as sticks. (**c**) Comparison of the putative glycerol exit route between PFL and hMGL in its open, semi-open, and closed conformation. Key residues lining the proposed exit pathway in hMGL. Residues of PFL and hMGL in the semi-open conformation are labeled in black and orange, respectively.

- Alcohol-binding pocket. The catalytic site is also well conserved between PFL, hMGL, and mtMGL. In particular, there is a high identity in the alcohol-binding pocket. In the PFL crystal structure, this pocket is bordered by the side chain of Glu24, Arg28, His86, Glu234, Tyr159, Leu149, Ile205, and Leu144, in addition to the Leu22 main chain and the catalytic triad residues Ser87 and His233 (Figure 4a and Figure S1). These residues likely accommodate the substrates by establishing key interactions with its glycerol moiety, which is in agreement with the glycerol or MPD molecules systematically found in the crystal structures of PFL, hMGL, or mtMGL enzymes. These are extremely well conserved in hMGL and mtMGL, with the exception of PFL Glu234, which is involved in a salt bridge with Arg28, which is also present in mtMGL but is replaced by the hydrophobic Val280 in hMGL.
- *Acyl-binding pocket site*. In the absence of a crystal structure of PFL—or its homologs—in complex with a substrate or a substrate-derived ligand, it is difficult to precisely map the acyl-binding pocket. However, the presence, in the PFL active site, of a co-crystallized detergent molecule with a lauryl chain moiety (Figure S3) allows to delineate this pocket and to identify the residues likely involved in substrate recognition. As expected, the PFL pocket is mainly hydrophobic and bordered on the one side by Asn142, a Thr120 side chain, and a Ser141 main chain and, on the other side, by the side chain of residues Leu22, Leu88, Val177, Phe178, and Ile169 (Figure 4b). Then, the lipid chain projects toward a hydrophobic patch in the lid made of Met124, Leu127, Leu131, Leu138, and Leu140 side chains. Whilst the homology is lower than in the alcohol-binding pocket, several residues of the acyl-binding pocket are also conserved between PFL and hMGL or mtMGL (Figure S1).

#### 3.1.3. PFL Substrate Preference

**Figure 5:**
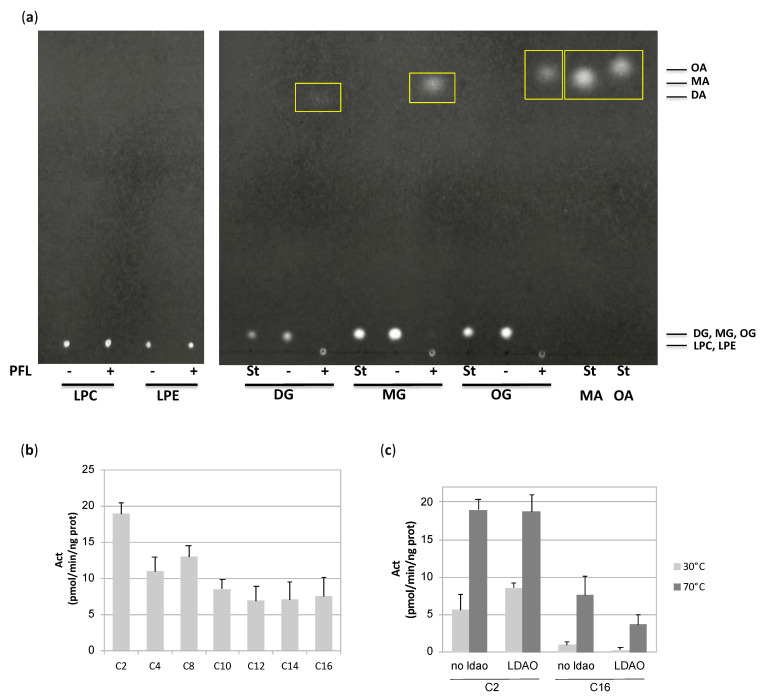
Measurement of PFL lipase activity. (**a**) PFL lipase activity against lysophosphatidylcholine (LPC), lysophosphatidylethanolamine (LPE), decanoyl glycerol (DG), myristoyl glycerol (MG), and oleoyl glycerol (OG). After incubation, the lipids were separated by thin layer chromatography and revealed using primuline. Monoacylglycerols, myristic acid, and oleic acid were used as standards (St). Hydrolysis products are indicated by a yellow frame. On the contrary to lysophospholipids, the incubation of monoacylglycerols in the presence of PFL yielded to a complete disappearance of the substrate, coupled to the formation of the corresponding fatty acid. (**b**,**c**) Hydrolase activity on *p*-nitrophenyl ester derivatives of acyl chain length ranging from C2 to C16 at 70 °C (**b**) or 30 and 70 °C (**c**). The amount of generated product was calculated after measuring the absorbance at 445 nm and blank subtraction. Values are the mean ± standard deviation (*n* = 3–11). In (**c**), the reaction was performed in the absence or the presence of LDAO 0.1%.

PFL was annotated as a lysophospholipase, as well as TBL, with which it shares 73% identity and 87% similitude (BioCyc database, access numbers G1BRO-699 and TERMP_01081). However, given the structural homology of the alcohol-binding pocket and the lid compared to hMGL and mtMGL, it would be unlikely that the enzyme is active on lysophospholipids rather than monoacylglycerols. Moreover, due to its limited size, there is little chance that the alcohol-binding pocket allows the binding of the rather bulky polar head group of lysophospholipids or phospholipids. Therefore, we tested PFL for its ability to hydrolyze lipids with various polar head groups. As expected, PFL failed to hydrolyze lysophosphatidylethanolamine or lysophosphatidylcholine. On the contrary, an activity was detected on decanoyl- as well as myristoyl- and oleoyl-glycerol (Figure 5a). We also measured the activity of the enzyme on *p*-nitrophenyl esters substrates of various acyl chain lengths and showed that PFL is active on substrates ranging from C2 to C16 (Figure 5b).

The ability of methyl arachidonyl fluorophosphonate (MAFP), a long-chain substrate-like covalent inhibitor of hMGL, to inhibit PFL was measured. A strong inhibition of the hydrolysis of *p*-nitrophenyl acetate was observed with a pIC50 of 7.82. The ability of MAFP to interact with PFL was further evidenced by a thermofluor assay, in which the binding of a ligand is evidenced through the measure of the thermal unfolding transition of the protein (melting temperature, Tm). As ligands typically result in a stabilization of the folded state of the protein, the binding event is often accompanied by an increase of the temperature at which the protein unfolds [38]. In the presence or absence of detergent micelles, the apo form and the MAFP-bound enzyme are characterized by different Tm values (Table 2). In the absence of micelles, the poorly defined melting temperature curve of the apoenzyme improves drastically when MAFP is added. A melting temperature of 89.9 °C was measured in the presence of the inhibitor, which is the highest temperature observed for PFL. In the presence of detergent, the apo- and inhibitor-bound enzymes have Tm values of 76.4 °C and 83.5 °C, respectively.

Based on molecular modeling studies and on the crystal structure of open and closed states of hMGL and bMGL, the existence of a glycerol exit route was proposed, whose access is controlled by the gatekeeper residue Ile179 or Ile145 for the open human and bacterial enzyme, respectively [7,17,26,36]. However, no hole is observed in the wall of PFL active site at this place, similarly to what is observed for mtMGL (Figure 4c). Indeed, in PFL, the residue Leu144, which corresponds to the gatekeeper residue in hMGL, locks the aperture compared to the fully open hMGL and actually lies in an intermediate state between the semi-open and closed human enzyme. Moreover, Asn142, and to a lesser extent Pro146, also contribute to restricting the access. Thus, in the crystal structure of PFL, such a hypothesized exit route for glycerol is seen to be closed.

#### 3.1.4. Lid Plasticity

Many lipases have evolved to control the access to their active site by conferring to the lid the ability to alternate between a closed and an open state [9]. Regarding MGLs, such plasticity has been evidenced experimentally for hMGL [7,25,26], bMGL [36], as well as scMGL [35]. For mtMGL [8] and PFL, only one crystal structure has been obtained to date. In the case of hMGL, it was reported that the binding to a lipid interface triggers enzyme activation [39]. Therefore, we tested the ability of a detergent to enhance PFL activity. No increase of C2 and C16 p-nitrophenyl esters hydrolysis was observed at 30 or 70 °C (Figure 5c). For the C2 compound, various detergent concentrations were tested, ranging from 0.25 to 2 times the detergent critical micellar concentration, without leading to a modification of the activity (Figure S6).

Temperature was also reported to mediate the opening of *Pyrococcus furiosus* PF2001 esterase through dimerization and complete remodeling of cap subdomain [40]. Thus, we tested the PFL activity on a short and a long chain *p*-nitrophenyl derived substrate at low and high temperatures (Figure 5c). On the C2 derivative, the activity is 2 to 3-fold higher at 70 °C compared to 30 °C. Whilst PFL is only barely active on the C16 derivative at the lower temperature, a substantial increase of the activity is observed at 70 °C. This indicates that the temperature increase could contribute to the wider opening of PFL needed to accommodate the substrates.

To assess the size of the PFL active site aperture, we measured the distance between the Cα of Leu140, located in the linker H56, and the center of mass of residues 121–131, in the H5 helix. This distance decreases dramatically during the closing of hMGL, from 13.3 Å to 8.4 Å. Although slightly smaller in PFL, with a value of 11.9 Å, it is very close to that of the open conformations of hMGL or mtMGL (12.9 Å). The same observations were made when considering the distance between the Cα of residues Phe123 and Leu140, which will be used later in this work to assess the opening state of PFL (Figure S7). More precise distance descriptors are presented further in the text when reporting MD results. Other arguments are also in favor of PFL being in an open state. First, as discussed above, the PFL lid and hinges’ overall organization resembles that of mtMGL and hMGLs in their open conformation. Second, hydrophobic, and not hydrophilic, residues are solvent-exposed at the surface of the lid, similarly as in open hMGL and mtMGL. Third, the opening created by the lid seems wide enough to allow the passage of the substrate. Taken together, we hypothesized that the obtained structure constitutes an at least partially open state. However, to gain further understanding of a potential conformational plasticity of the lid and to highlight the structural elements that favor the conformational states, ENM calculations and MD simulations were carried out.

### 3.2. In Silico Results

#### 3.2.1. Deformability of the Monoglyceride Lipase Structure

The iMODS predicted B factors are displayed in Figure S8 together with the corresponding experimental profile of the Cα atoms. The calculated B factor profile more clearly delineates the enzyme sequence into small segments compared to the experimental profile, and two highly mobile sections are observed. They include Ala113 to Ser141 and Ser150 to Arg168, which contain H5 and H7, respectively (Figure S8a). The two helices actually move in a correlated fashion as visualized by the black arrow on the Cα covariance map displayed in Figure S8b. Other correlated motions are detected between the strands of a β-sheet involving sequences 100–120 and 190–210 as well as spatially close sequences 50–70 with 160–180, which shape a part of the enzyme substrate-binding pocket (green and blue arrows in Figure S8b, respectively). Except for 100–120 and 190–210, these regions constitute or are at the vicinity of the lid or hinges or the proposed glycerol exit route in homologous MGLs.

Of the identified highly mobile zones, regions 113–141 and 160–180 correspond to the H5 helix encompassed by the two hinges and a part of the H8 helix, respectively. Interestingly, during the course of this study, we also crystallized the homologous enzyme TBL. In TBL, whilst H5 and its hinges, as well as H8 and H9, are well defined and completely superimposable with PFL in the chain A of the dimer, they are characterized by high flexibility in chain B. Indeed, these regions displayed co-existing alternate conformations or high disorder. Unfortunately, modeling this apparent flexibility was revealed to be very challenging, impeding the finalization of the structure (high R-free value).

As discussed earlier, the Cα123-Cα140 distance can be used to describe the conformation state of the enzyme. These two specific Cα are characterized by a large and low normalized mobility value of 0.923 and 0.497, respectively (Figure S8a).

The ENM modeling does not include finer interaction effects such as those involving a ligand and the solvent. MD simulations were carried out to more deeply investigate the properties of a confined ligand interacting within the enzyme substrate-binding pocket, the effect on the pocket aperture, and to suggest a structural classification of the experimental crystal structure of the enzyme.

#### 3.2.2. Overall and Atomic Fluctuations

The MD analysis was performed with and without glycerol and LDAO, which are present in the crystal structure, as well as in the presence of the covalent ligand MAFP instead of LDAO. In the case of the systems with LDAO, the protein undergoes more important conformational changes than for the other systems during the 300 ns MD trajectory. Thus, the analyses were achieved over the last 200 ns only. The resulting Root Mean Square Displacement (RMSD) profiles versus the initial system conformations are given in Figure S9.

The Root Mean Square Fluctuation (RMSF) profiles of the Cα atoms are reported in Figure S10. As already observed from the ENM calculations, the helices H5 and H7 (Table S2) are characterized by the largest fluctuations, especially in the presence of glycerol and LDAO (Figure S10c). However, their magnitude is reduced in the presence of MAFP, which further supports the increased stability observed in the thermofluor assay in the presence of this inhibitor. In this system, the highest RMSF value is observed around the residue Ala188 (Figure S10d) and is associated with a deconstruction of the extremity of helix H8 compared to the initial crystal structure. Noteworthy, in the chain B of the TBL structure, the corresponding residues 187–190 are also highly disordered, together with a deconstruction of the helix H9 and high mobility of H8. Significant changes in the RMSF profile occur for PFL when the temperature is set to 70 °C. The enzyme lid, as well as the sequence 211–224 which includes the helix H10, show enhanced displacements (Figure S10a). Particularly, the RSMF is increased around residues 115 and 137, which are in the hinge regions of the H5 helix. Contrarily, the residues 19–22, 49–55, and 147–169, which involve the helices H6 and H7 of the alcohol-binding pocket, have a reduced mobility as a consequence of its tighter closure with, e.g., a Cα31–Cα153 distance value of 1.281 ± 0.063 nm to be compared to 1.951 ± 0.103 at 27 °C. On the whole, the three-dimensional structure of PFL appears to be stable under an increase of temperature; nevertheless, there is also an enhanced mobility of the lid and a reduced mobility of alcohol-binding pocket residues.

#### 3.2.3. Influence of the Ligand Conformations on the Enzyme Opening

The Cα123–Cα140 and Cα124–Cα142 distances were investigated to study the putative opening or closing of the enzyme and were shown to decrease in comparison with the crystal structure in all the simulations (Table 3, Figure S11A,B), with the highest and lowest values respectively measured at 70 °C and for the simulations performed without any ligand (LDAO or MAFP) in the acyl-binding pocket. A strict comparison of the PFL-GL simulation results with the corresponding crystal structure values remains difficult (due to the simulation conditions, e.g., the effect of the selected FF, the chemical composition of the enzyme surroundings). However, the mean distance values show that the ligands participate in the enlargement of the substrate-binding pocket aperture, thus suggesting that the crystal enzyme structure is not in its most closed conformation and is stabilized by interactions with the ligand, as illustrated further in the text.

On the whole, several movements restrict the accessibility to the active site by bringing H5 and its *N*-terminal hinge closer to the linker H56. In PFL-G, which is characterized by short Cα123–Cα140 and Cα124–Cα142 distances, an inward motion of the *N*-term hinge of H5 is observed, which triggers an inward translation of the *N*-term end of H5. A translation of the linker H56 toward H5 is also detected. In particular, the side chain of Ser122 can occasionally be hydrogen-bonded to the backbone of Asn142, in addition to a more frequent hydrogen bond formed between the side chain of Asn142 and the backbone of Met124 (Figure S12A). The sharpest transitions observed in the Cα123–Cα140 profile at *t* = 270 ns (Figure S11A) and in the Cα124–Cα142 profile at 298 ns (Figure S11B) of the system PFL-G are due to a deconstruction of the hydrogen bond pattern (Figure S13b).

For PFL-GL, the Cα124–Cα142 distance profile illustrates sharper transitions than the Cα123–Cα140 one (Figure S11A,B, respectively). Particularly, the low mean value of 0.831 ± 0.072 nm observed in the Cα124–Cα142 profile from 100 to 142.8 ns, is well correlated to the presence of two hydrogen bonds formed by Ser117 with LDAO and Ile204 (Figure S12B). After 142.8 ns, the Cα124–Cα142 distance increases up to a mean value of 1.138 ± 0.088 nm, while Ser117-LDAO hydrogen bonds rarely occur. Starting at *t* = 255 ns, the enzyme structure forms a single hydrogen bond with LDAO, and the Cα124–Cα142 distance is lowered to a mean value of 1.029 ± 0.045 nm (Figure S11B).

In PFL-GM, the distance profiles are relatively stable, except for occasional and short-lived transitions. Indeed, the Ser117 and Asn142 hydrogen bond profiles also show a steady behavior (Figure S12C). A long-lived Ser117–Asn142 hydrogen bond is also observed at 70 °C (Figure S12D). It is only when such a hydrogen bond is broken, between *t* = 206 and 218 ns, that the Cα123–Cα140, the Cα124–Cα142, but also the Cα122–Cα204 distances, increase (Figures S11A,B and S12F, respectively). Asn142 is also constantly hydrogen bonded to Thr120, except for a very short time lapse, between 218 and 219 ns. It is followed by the sudden decrease in the Cα122–Cα204 and Cα124–Cα142 distance values, which corresponds to the reoccurring of the Thr120–Asn142 hydrogen bond.

**Figure 6:**
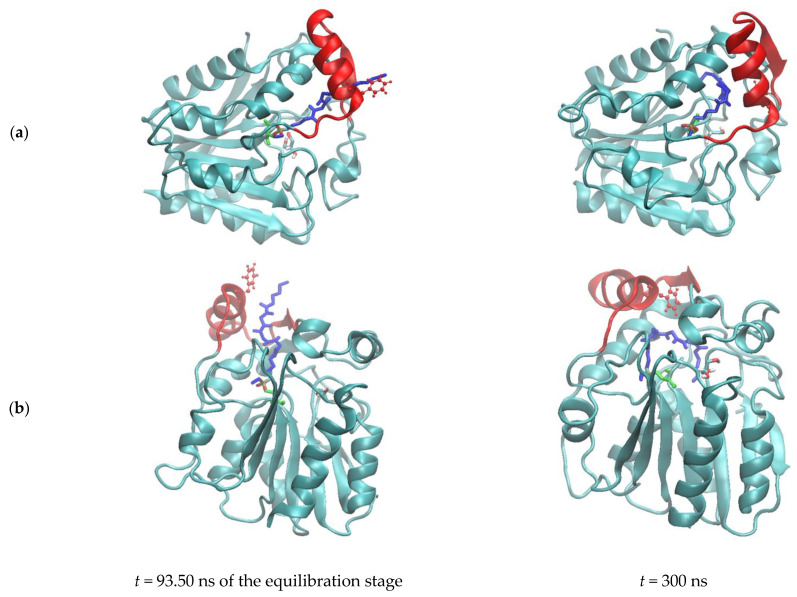
Snapshots of the Molecular Dynamics (MD) simulation of PFL-GM (cyan) at 300 K and 1 bar. The PFL lid is in red. MAFP, glycerol, and Ser87 are displayed using blue, atom-types colored, and green sticks, respectively. Phe123 is shown using red balls and sticks. (**a**) Side view, (**b**) bottom view.

**Figure 7:**
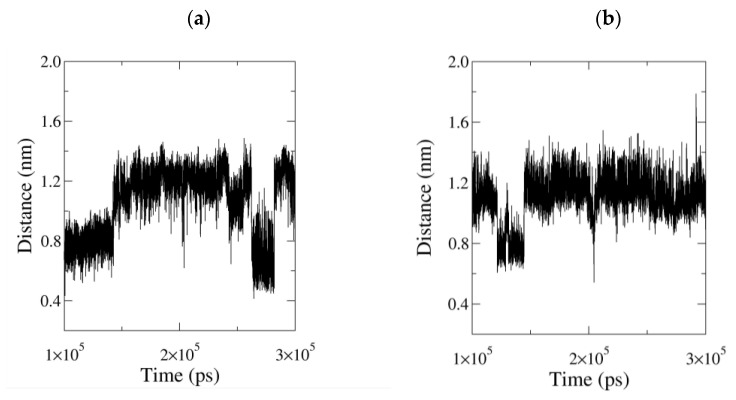
(**a**) LDAO distance profile N_LDAO_-C1_LDAO_ and (**b**) methyl arachidonyl fluorophosphonate (MAFP) distance profile P-C26, as obtained from the last 200 ns of the MD simulation of PFL-GL and PFL-GM, respectively, at 300 K and 1 bar. The crystal structure with a value of 1.183 nm in the case of LDAO adopts an extended conformation.

**Figure 8:**
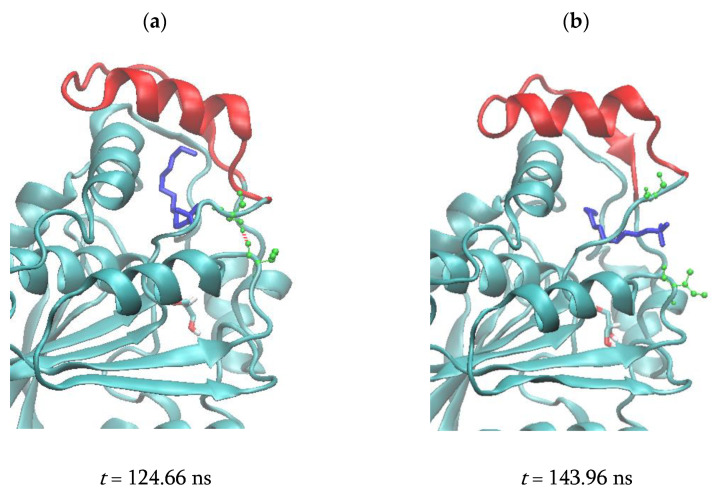
Snapshots of the MD simulation of PFL-GL (cyan) at 300 K and 1 bar. The PFL lid is in red. LDAO and glycerol are displayed using blue and colored sticks, respectively. Ser117 and Ile204 are represented using green balls and sticks. (**a**) The hydrogen bond is depicted with red dashes. (**b**) The Ser117-Ile204 hydrogen bond is broken.

The ligands LDAO and MAFP undergo several conformational changes, adopting either folded or extended configurations (Figure S14 and Figure 6, respectively). Their geometry is monitored using the distance value between their end atoms, i.e., the N_LDAO_-C1_LDAO_ and P-C26 distance, respectively (Figure 7). During the simulation of the system PFL-GL, LDAO alternates between folded and extended conformations (Figure 7 and Figure S13). In the extended conformation, e.g., from 142.8 to 262.4 ns, its polar head crosses the pocket limit at the level of residues Ser117 and Ile204 (Figure 8).

In the crystal structure of PFL, as well as during the first 142.8 ns of the MD trajectory of PFL-GL, both residues 117 and 204 are hydrogen bonded and thus act as a constraint to a change in the H5 position. Later, the hydrogen bond constraint is released and H5 is shifted away, toward a wider opening of the lid (Figure S12E), consistently with the lack of any of such hydrogen bond in the open form of the hMGL (pdb code 3hju [7]). The displacement of the helix H5 upon the breaking of the Ser117–Ile204 hydrogen bond is evaluated through the Cα122–Cα204 distance profile (Figure S12F). Such a displacement takes place around *t* = 142 ns, when the hydrogen bond is broken, and it temporarily reaches the amplitude of 2.49 nm at *t* = 188.14 ns (Figure S12G). Then, a relaxation occurs around *t* = 250 ns. In PFL-G, the increase of the Cα122–Cα204 observed around 250 ns is correlated with a change in the hydrogen bond pattern formed by Asn142 (Figure S13a). Before 250 ns, Asn142 is mainly hydrogen bonded to residues Ser122, Phe123, and Met124, while after 250 ns, it is bonded to residues Lys119 and Thr120. Thus, the Ser117-Ile204 hydrogen bond is seen here as a structural feature that helps to stabilize the closed form of the enzyme. Even though the Ser117-Ile204 hydrogen bond is present in the crystal structure of the enzyme, the experimental conformation is characterized by rather large Cα123–Cα140 and Cα124–Cα142 distances due to the absence of any hydrogen bond occurring between the helix H5 and its facing residues in the lid.

As mentioned earlier, Asn142 of the lid can form hydrogen bonds with facing residues such as Thr120, Ser122, Phe123, and Met124. It is observed in the system PFL-G and, to a lesser extent, in PFL-GL (Figure S13). In PFL-GM, the constraints imposed by the arachidonyl chain of the inhibitor impedes the onset of such interaction. Particularly, when MAFP adopts a folded conformation, the hydrophobic end of MAFP is oriented toward the lid, as observed between 121 and 144 ns (Figure S15). Additionally, the presence of the covalent bond between MAFP and PFL induces a deconstruction of the helix H4, at the level of residues 88 to 90. Such a deconstruction limits the space available to the ligand and prevents H5 from forming a hydrogen bond with Asn142. In PFL-G, these hydrogen bonds strongly limit the extend of the lid opening and reduce the Cα123–Cα140 distance to 0.764 ± 0.120 nm.

Regarding the ligand MAFP covalently bound to the enzyme, it appears to stay located within the substrate-binding pocket. Nevertheless, whilst the Cα123–Cα140 fluctuations are not associated with an opening of the lid, the crossing of the ligand head through the lid remains possible. Such an event has been observed only once during the equilibration stage of the PFL-GM simulation (Figure 6 left). During the crossing, the side chain of Phe123 is flipped away from the opening and leaves a free passage to MAFP. Later, the phenyl ring flips inward and, together with a movement of H5, blocks the aperture (Figure 6 right). Phe123 finds its equivalent as Phe159 in the structure of hMGL [17]. Through MD simulations, Riccardi et al. identified Phe159 as a pulling residue that might control the insertion of the substrate. In their work, the side chain of Phe159 can be oriented toward the lipid bilayer, similarly to Figure 6 (left), or toward the enzyme, similarly to Figure 6 (right). These conformational changes might be favored due to the flexibility of MAFP, for which two main conformations are identified within the substrate-binding pocket (Figure 7 right). They are characterized by P-C26 atom–atom distances of about 0.8 and 1.2 nm, while the extended crystal structure has a value of 1.183 nm.

In the fully extended conformation observed during the equilibration stage, illustrated in Figure 6 left, the distance P-C26 is equal to 2.153 nm. That short-lived crossing of the MAFP through the enzyme surface involves negative values of the N-Cα-Cβ-Cγ angle of Phe123; i.e., Phe123 points outwards from the acyl-binding pocket, and the Cα123–Cα140 distance increases (Figure S16).

#### 3.2.4. Correlated Lid Motions

A Principal Component Analysis (PCA) of a covariance map allows determining the Principal Component (PC), the first of them characterizing the motion with the largest amplitude [41]. In addition to the hereabove detailed description of the lid closure elements, covariance analyses were carried out to generate a global view of the enzyme mobile moieties, giving information about their amplitudes and how they are correlated.

To easily determine which protein deformation is characterized by the first *PC*, i.e., which Cα–Cα distance is associated with the first *PC*, correlation maps are calculated using:

(1) $$\kappa_{ij}=\left[\frac{1}{N_{f}}\overset{N_{f}}{\underset{n=1}{\sum }}\left(PC_{n}\;d_{ij,n}\right)-\bar{PC}\;\bar{d_{ij}}\right]\frac{1}{\sigma_{PC}\;\sigma_{ij}}$$

where *N_f_* is the number of time frames considered in the calculation, and $\bar{X}$ and *σ_X_* stand for the mean and standard deviation of *X*, respectively. Then, each absolute value of *κ* is multiplied by the largest range of the corresponding Cα-Cα distances, ∆*d* = *d_max_* − *d_min_*, so as to visually emphasize large amplitude motions that are correlated with the first PC. For the *apo* form of the enzyme, the PC1-based |*κ*∆*d*| map clearly shows that the distances involving the residues 162 to 164 are highly fluctuating and correlated to the first PC (Figure S17a). Residues 135 to 140 and 178 to 182 are involved to a lesser extent. All these residues do not belong to the lid of the enzyme as confirmed by the modeled corresponding motion displayed in Figure S18a. It is consistent with the stable distance profiles of the enzyme stressed in Figures S11A,B and S12F. Contrarily, large amplitude motions of the lid residues are detected for the systems PFL-G, PFL-GL, and PFL-70. In PFL-G, residues 121 to 123 are the most mobile versus the rest of the enzyme, which confirms the use of Cα123 as a descriptor of the lid conformation (Figures S17b and S18b), while in PFL-GL, a larger segment of the lid is involved (Figures S17c and S18c). At 70 °C, the strongest mobility is observed around residues 136–138 and 218–220, at the extremity of the H5 and H10 helices, respectively, consistently with the RSMF profile described earlier. As reported in Section 3.2.2, increasing the temperature actually favors the mobility of the entire lid (Figure S18e). Contrarily, the first PC of the covariance map shows that the lid of PFL-GM is only weakly mobile (Figures S17d and S18d), which is again in line with the stabilization of PFL observed experimentally upon MAFP binding. Rather, helices H8 and H9, also highly mobile in TBL structure, are the most correlated with the first PC. Thus, it appears that the presence of the ligand can play a role in the motion of the lid, as already suggested by Chen et al. from their MD studies on the hMGL [16]. Figure S18 also suggests a variety of lid deformation modes, i.e., with the largest displacements affecting the *N*-term hinge of the helix H5 (Figure S18b), the H5 helix moeity (Figure S18c), or the whole lid (Figure S18e), which explains the use of several distance parameters as lid-opening descriptors.

## 4. Discussion

Despite a limited sequence identity with hMGL or mtMGL, PFL displays a high level of structural conservation with these enzymes. The ligand binding pocket is lined by well-conserved residues, which explains the PFL substrate preference for monoacylglycerols compared to other lipids, particularly lysophospholipids. The proposed glycerol exit route is locked by Leu144, which corresponds to the gatekeeper residue Ile179 in hMGL and lies in an intermediate state between the open and closed states of the human enzyme [17]. In the present study, we highlight Phe123 as a residue is involved in the closure of the PFL lid, which mirrors the corresponding pulling residue identified earlier for the hMGL. We also identify a hydrogen bond involving Ser117 with Ile204 as well as Asn142 as either stabilizing or closure elements of H5. Particularly, the Ser117–Ile204 hydrogen bond is observed in the presence of the LDAO ligand, and Asn142 can play a closure function even at high temperature.

Several pieces of evidence highlight the strong flexibility of the PFL lid. The H5 helix, which constitutes the core of the cap, its hinges, but also the H7 and H8 helices, located at the close vicinity, are the most flexible parts of the enzyme. Beyond hMGL and mtMGL, such flexibility was observed experimentally for scMGL and bMGL earlier, as well as during our recent efforts to solve the structure of TBL, which is a close homolog of PFL [8,16,35,36,39].

The presence of LDAO or MAFP appears to limit the closure of the lid compared to the glycerol alone, which leads to the most closed lid conformation. Glycerol, LDAO, but also a high temperature, favor large amplitude motions of the lid, while MAFP stabilizes it. A stabilization of free and detergent-associated PFL was also observed experimentally upon MAFP binding. From our MD study, even if the presence of the inhibitor also leads to a closure of the lid compared to the crystal structure, it stabilizes a conformation that is less closed compared to the presence of glycerol alone, which is in agreement with the absence of a hydrogen bond between Asn142 and its facing residues when the inhibitor is bound. A comparison of the van der Waals surface of the crystal structure of PFL, and a closed structure observed for the PFL-G system, allows emphasizing the partial opening of the crystal structure (Figure S5A,B, respectively). Figure S5A also reports that the lid of the crystal structure of PFL is seen as partially open when a spherical probe with a radius size of up to 2.1 Å only is used, while the residues Leu144 and His166 delineate a very small aperture toward the alcohol-binding pocket that is not accessible to the solvent.

In the case of the hMGL, a synthetic inhibitor named “C2” stabilized a closed conformation characterized by a rotational and elongational movement of H5, together with a burying of the hydrophobic residues toward the interior of the protein [26]. However, a similar mechanism is unlikely to explain the stabilizing effect in PFL. First, our MD study does not highlight a similar movement of H5, which is in agreement with the presence of Pro121 in the *N*-term hinge of the helix that is absent from the human enzyme and likely prone to impose torsion restraints to PFL (Figure 3a). Second, the inhibitor does not impede the formation of higher molecular weight aggregates observed in the absence of detergent, which argues against the possibility of hydrophobic residues being hidden upon MAFP binding (not shown). In addition to the MAFP-associated stabilization of PFL, other observations collected during the crystallogenesis work constitute indications that ligand binding is determinant for the dynamics of the PFL lid. Indeed, crystals of PFL could not be obtained in the absence of LDAO or, on the contrary, in the presence of MAFP, which suggests that this inhibitor stabilizes an alternate conformation compared to the crystal form. It is noteworthy that despite extensive work conducted on the human enzyme after the elucidation of the structure of the apoenzyme, we also failed to crystallize hMGL in complex with MAFP.

Another question of interest is the relevance of a lipid interface activation mechanism for PFL. Whilst the activity of the hMGL is subject to interfacial activation, this phenomenon was not observed on PFL. Nevertheless, micellar concentrations of LDAO impede the association of the dimer toward higher oligomeric forms and allow the crystallization of the enzyme. We can hypothesize either that the detergent shields hydrophobic residues without necessarily leading to a complete lid opening or that the interaction with LDAO micelles locks the lid in a conformation that is suboptimal for substrate recognition or for the completion of the catalytic cycle.

Earlier, during the study of bMGL, researchers crystallized the enzyme in complex with various lipids and did not observe a clear relationship between the presence of these ligands and the lid conformational state, thus highlighting the stochastic character of the lid dynamics [36]. Along this line, we pointed out the temperature dependence of the activity, especially on substrates with longer acyl chain, on which PFL is inactive at 30 °C. This could be an indication that rather than on interfacial activation, the dynamics of the lid rely at least partially on the temperature, which is substantiated by the observations made in the theoretical study.

## 5. Conclusions

The crystal structure of PFL reveals a remarkable structural conservation with other MGLs, in particular the hMGL and mtMGL ones. Several structural characteristics, which may likely constitute key features of MGLs, are particularly well conserved between these enzymes. In agreement with these observations, PFL exhibits a lipase activity on various monoacylglycerols.

Due to the specific hydrogen bond, the closing/opening motions are hardly observed starting from closed forms of the enzyme in water, similarly to previous MD studies achieved on several lipases [14,15]. A comparison with the experiment remains difficult due to the approximation level of the FF and the enzyme surroundings. Nevertheless, the reported distances, in particular Cα123–Cα140, show that the active site entry is narrower compared to the crystal structure. The comparisons between the various MD simulations highlighted several lid stabilizing or closure elements, i.e., a Ser117–Ile204 hydrogen bond as well as hydrogen bonds formed between Asn142 and its facing amino acid lid residues. Riccardi et al. identified hMGL Ile179 as a gatekeeper residue that holds the substrate within the binding site [17]. In the present study, besides Leu144, which locks the glycerol exit route, we also identify Asn142 as a closure residue. As in other theoretical works, when flipped in the inward position, the lid residue Phe123 that corresponds to the hMGL Phe159 pulling residue appears to act as a blocking agent of the lid aperture due to its interaction with MAFP [35]. At high temperature, the enzyme structure remains very stable, as suggested by the MD calculations. Only short residue sequences such as the lid appear to be characterized by larger motions. Simultaneously, specific residues around the glycerol binding pocket have a lower mobility.

The high flexibility and closure propensity of the lid observed in the MD study, even in the presence of LDAO, could constitute indications that the crystal packing contributes at least in part to the stabilization of the conformational state highlighted in the experimental structure. Based on the MD study, as well as on the structural analysis and the comparison with other MGLs, we suggest that the structure reported herein at least partially represents an open conformation of the lipase, which could be stabilized by the crystal packing or the ligand, and that a closure of the lid can occur, which is an event that may depend on the temperature or the absence of the ligand. The ultimate proof of the mobility of the cap and the in-depth understanding of the molecular mechanisms that drive these dynamics should nevertheless benefit from the future elucidation of other experimental structures with the lid in alternate conformations or in complex with various ligands.

## Figures and Tables

**Table 1:** Description of the systems simulated using Gromacs5.1.4 at 300 K or 343 K and 1 bar. All systems are built from the PFL chain A.

	Glycerol	Ligand	No. of SPC Water Molecules	Final Box Size (nm)
PFL	-	-	14,383	7.83904
PFL-G	X	-	14,472	7.84390
PFL-GL	X	LDAO	14,372	7.83805
PFL-GM	X	MAFP	14,379	7.83855
PFL-70	-	-	14,383	7.93524

**Table 2:** Thermofluor analysis of PFL and influence of the presence of detergent micelles and the inhibitor MAFP 50 µM. Tm values (°C) are the mean ± standard deviation of at least four experiments (n.d.: not determined, due to the poorly defined profile of the melting curve).

	/	MAFP
**/**	n.d.	89.9 ± 2.1
**LDAO 0.1%**	76.4 ± 1.8	83.5 ± 1.3

**Table 3:** Cα123–Cα140 and Cα124–Cα142 distance profiles calculated for the PFL structure, as obtained from the last 200 ns of MD trajectories at 300 K or 343 K and 1 bar. The reported values are the mean ± standard deviation.

	Cα123–Cα140 Distance (nm)	Cα124–Cα142 Distance (nm)
PFL	1.061 ± 0.071	0.722 ± 0.063
PFL-G	0.764 ± 0.120	0.779 ± 0.112
PFL-GL	1.014 ± 0.061	1.048 ± 0.144
PFL-GM	0.922 ± 0.076	1.006 ± 0.061
PFL-70	1.240 ± 0.117	1.147 ± 0.126
Crystal structure	1.493	1.076

## Data Availability

The structure of PFL was deposited in the Protein Data Bank (PDB) (pdb code 6qe2).

## Supplementary Materials

The following are available online at https://www.mdpi.com/article/10.3390/biom11040533/s1, Figure S1: Sequence alignment and identity of PFL with other MGLs; Figure S2: topology diagram of PFL; Figure S3: Electron density maps in PFL active site; Figure S4: Overlay of the structures of PFL, hMGL, and mtMGL; Figure S5A. Accessible surface of the crystal PFL chain A; Figure S5B. Electrostatic potential surface of the PFL; Figure S6: PFL activity in the presence of LDAO; Figure S7: Comparison of the opening state of PFL, hMGL, and mtMGL; Figure S8: (a) iMODS and experimental B factor profiles of PFL, (b) iMODS Cα covariance map; Figure S9: RMSD profiles of MD trajectories. Figure S10: Cα RMSF profile of the MD trajectories; Figure S11A,B: Cα123–Cα140 and Cα124–Cα142 distance profiles obtained from the MD trajectories; Figure S12A: Snapshot of the system PFL-G; Figure S12B-D: Hydrogen bonds formed by Ser117 and Asn142 as obtained from the PFL-GL, PFL-GM, and PFL-70 MD simulations; Figure S12E: Superimposition of two conformations obtained from the PFL-GL MD trajectories; Figure S12F: Cα122–Cα204 distance profiles obtained from the MD trajectories; Figure S12G: Snapshots for the system PFL-GL; Figure S13: Snapshots and hydrogen bond maps for the systems PFL-G and PFL-GL; Figure S14: PFL structures as obtained from the crystallographic experiment and at *t* = 0 ns and the end of the PFL-GL MD production stage; Figure S15: Snapshot of the system PFL-GM; Figure S16: MAFP distance profile P-C26, dihedral N-Cα-Cβ-Cγ of Phe123 in PFL-GM, and Cα123-Cα140 distance profiles for the system PFL-GM; Figure S17: |κ.Δd| maps calculated using the first PC of the Cα covariance matrix from the MD trajectories; Figure S18: PFL motion modeled using the first PC of the Cα covariance matrix, from the MD; Table S1: Data collection and refinement statistics; Table S2: Helix content of chain A of protein PFL; SM1: Technical details regarding the ligand building; SM2: Description of the Molecular Dynamics calculations.

## Abbreviations

AA	Amino Acid
ATB	Automated Topology Builder
bMGL	*Bacillus* sp. H257 MGL
ENM	Elastic Network Model
FF	Force Field
hMGL	human monoglyceride lipase
LDAO	Lauryldimethylamine oxide
MAFP	methyl arachidonyl fluorophosphonate
MD	Molecular Dynamics
MGL	Monoglyceride lipase
mtMGL	*Mycobacterium tuberculosis* monoglyceride Lipase
PCA	Principal Component Analysis
PDB	Protein Data Bank
PFL	*Palaeococcus ferrophilus* monoglyceride Lipase
RMSD	Root Mean Square Displacement
RMSF	Root Mean Square Fluctuation
scMGL	*Saccharomyces cerevisiae* monoglyceride Lipase
SPC	Simple Point Charge
VMD	Visual Molecular Dynamics
