# Supplementary material for: Structure and Dynamics of an Archeal Monoglyceride Lipase from Palaeococcus ferrophilus as Revealed by Crystallography and In Silico Analysis
Source: Biomolecules. 2021 Apr 3;11(4):533. doi: 10.3390/biom11040533 (PMC8065475; doi:10.3390/biom11040533)
Supplement: Supplementary file 1 [file biomolecules-11-00533-s001.pdf]

Supplementary Material

Structure and dynamics of an archeal monoglyceride lipase from *Palaeococcus ferrophilus* as revealed by crystallography and *in silico* analysis

Geoffray Labar G. [1,*], Nathalie Brandt [1], Amaury Flaba [1], Johan Wouters [2] and Laurence Leherte [2,*]

[1] Institut de Recherches Labiris, 1 Avenue E. Gryson, B-1070 Bruxelles, Belgium.
[2] Laboratoire de Chimie Biologique Structurale, Department of Chemistry, Namur Medicine & Drug Innovation Center (NAMEDIC-NARILIS), Namur Research Institute for Life Sciences (NARILIS), Namur Institute of Structured Matter (NISM), University of Namur, Rue de Bruxelles 61, B-5000 Namur, Belgium.

* Correspondence: glabar@spfb.brussels (G.L.); Laurence.leherte@unamur.be (L.L.)

(a)

```
hMGL          ---------MPEESSPRRTPQSIPYQDLPHLVNADGQYLFCRYWK-PT--GTPKALIFVS    48
PFL           --------------------------------------MELYRAKFG--TPERGWVVLV    19
TBL           ---------------------------------------MIYKAKFG--TPERGWIVLV    18
mtMGL         ---------MT------------TTRTERNFAGIGDVRIVYDVWT-PD--TAPQAVVVLA    36
scMGL         -----------MAPYPYKVQTTV---PELQYENFDGAKFGYMFWPVQNGTNEVRGRVLLI    46
BH257         MGSSHHHHHHSSGLVPRGSH------MSEQYPVLSGAEPFY---A-----ENGPVGVLLV    46
                                                                      :.:

hMGL          HGAGEHSGRYEELARMLMGLDLLVFAHDHVGHGQSEGE--RMVVSDFHVFVRDVLQHV--    104
PFL           HGLGEHSGRYGKLIELLNGAGFGVYAFDWPGHGKSPGK--RGHTSV-----EEAMKII--    70
TBL           HGLGEHSGRYEKLINMLVDEGFAVYTFDWPGHGKSEGK--RGHATV-----EQAMKII--    69
mtMGL         HGLGEHARRYDHVAQRLGAAGLVTYALDHRGHGRSGGK--RVLVRDISEYTADFDTLV--    92
scMGL         HGFGEYTKIQFRLMDHLSLNGYESFTFDQRGAGVTSPGRSKGVTDEYHVFND-LEHFVEK    105
BH257         HGFTGTPHSMRPLAEAYAKAGYTVCLPRLKGHGTHYEDMER---TTFHDWVAS----VEE    99
              **          :       .         * *       :                :

hMGL          DSMQKDYPGLPVFLLGHSMGGAIAILTAAER--PGHFAGMVLISPLVLANPESAT-TFKV    161
PFL           DSI-IEELGEKPFLFGHSLGGLTVIRYAETR--PDKIMGVVASSPALAKSP-KTP-SFMV    125
TBL           DEI-IEEIGEKPFLFGHSLGGLTVIRYAQTR--PDRIKGIIASSPALEKSP-KTP-SFMV    124
mtMGL         GIATREYPGCKRIVLGHSMGGGIVFAYGVER--PDNYDLMVLSAPAVAAQD-LVS-PVVA    148
scMGL         NLSECKAKGIPLFMWGHSMGGGICLNYACQGKHKNEISGYIGSGPLIILHPHTMYNKPTQ    165
BH257         GYGWLKQRCQTIFVTGLSMGGTLTLYLAEHH--P-DICGIVPINAAVDIPA--------I    148
              .    .      :: * *:**   :  .            :     :

hMGL          LAAKVLNLVLPNLSLG-PIDSSVLSRNKTEV-DIYNSDPL----------ICRAGLKVCF    209
PFL           ALAKVLGRITPGLSLSNGLDPKLLSRNPDAV-KRYIEDPL----------VHD-RISGKL    173
TBL           LLAKVLGSIVPTLTLSNGIDPNLLSRNKEAV-RKYVEDKL----------VHD-KISAAL    172
mtMGL         VAAKLLGVVVPGLPVQ-ELDFTAISRDPEVV-QAYNTDPL----------VHHGRVPAGI    196
scMGL         IIAPLLAKFLPRVRIDTGLDLKGITSDKAYR-AFLGSDPMSVPLYGSFRQIHDF---MQR    221
BH257         AAGMTGGGELPRYLDSIGSDLKN----PDVKELAYEKTPT------------------AS    186
                .       *        * .

hMGL          GIQLLNAVSRVERALPKLTVPFLLLQGSADRLCDSKGAYLLMELAKSQDKTLKIYEGAYH    269
PFL           GMSVFDNMERAHKEAERIKAPVLLLVGTADIITPPEGSRRLFEELKVKDKTIMEFKGAYH    233
TBL           GKSIFENMEKAHEDAEKVKVPILILIGTEDVITPPEGARKLFENLTVEDKMLKEFKGAYH    232
mtMGL         GRALLQVGETMPRRAPALTAPLLVLHGTDDRLIPIEGSRRLVECVGSADVQLKEYPGLYH    256
scMGL         GAKLYKNENNYIQKNFAKDKPVIIMHGQDDTINDPKGSEKFIQDCPSADKELKLYPGARH    281
BH257         LLQLARLMAQTKAKLDRIVCPALIFVSDEDHVVPPGNADIIFQGISSTEKEIVRLRNSYH    246
                 :                * ::: .  * :    .:  :.:     :  :    .  *

hMGL          VLHKE-LPEVTNSVFHEINMWVSQRTATAG-TASPPLEVDLQGDHGLSAWSHPQFEK     324
PFL           EIFED-PEW-GEEFHRAIVEWLVSHSRGDLEWASAERASSIMGD-------------     275
TBL           EIFED-PEW-GDEFYMTIIEWLRIHS-------------------------------     256
mtMGL         EVFNE-PE--RNQVLDDVVAWLTERL-------------------------------     279
scMGL         SIFSLETDKVFNTVFNDMKQWLDKHTTTEAKP-------------------------     313
BH257         VATLDY-D--QPMIIERSLEFFAKHAG------------------------------     270
                           .      :.  :
```

(b)

	Whole sequence	Cap
hMGL	100	100.00
PFL	32.4	38.9
TBL	33.1	38.9
mtMGL	33.7	30.9
scMGL	23.8	17.4
BH257MGL	18.5	13.3

Figure S1. (**a**) Clustal Omega alignment of PFL and TBL with monoglyceride lipases of known tri-dimensional structure [1]. hMGL: human MGL; mtMGL: *Mycobacterium tuberculosis* MGL; scMGL: Saccharomyces cerevisiae S288C MGL; BH257: *Bacillus sp.* H257 MGL. The cap region is highlighted in gray and the residues belonging to the catalytic triad are in bold. The residues lining the PFL alcohol- and acyl-binding pockets are colored in red and blue, respectively. (**b**) Amino acid sequence identity based on the whole sequence or the cap region, as defined in Riegler-Berket et al. (residues 150-205 of hMGL) [2].

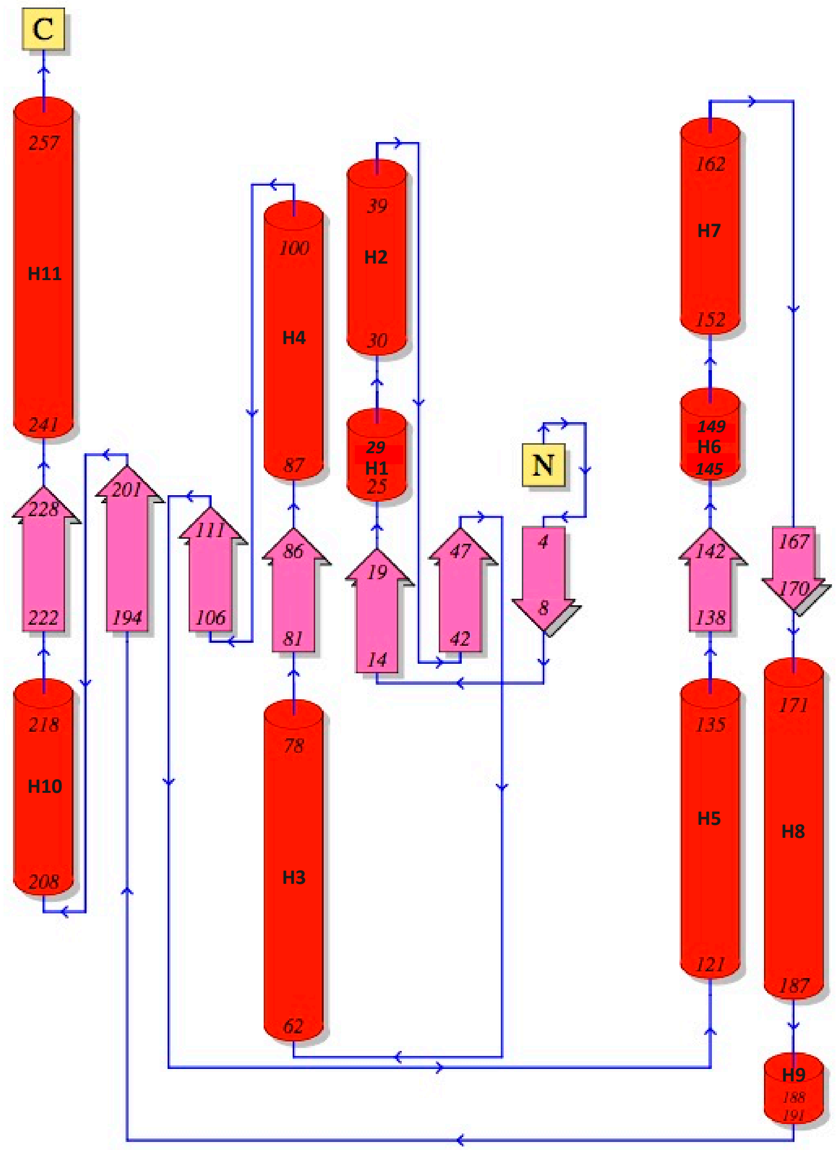

Figure S2. 2D topology diagram of PFL, as obtained from PDBSum software [3]. The position of the helices is indicated. The linker H56 refers to the region connecting H5 to H6 and comprises residues 135-144.

(a)

(b)

(c)

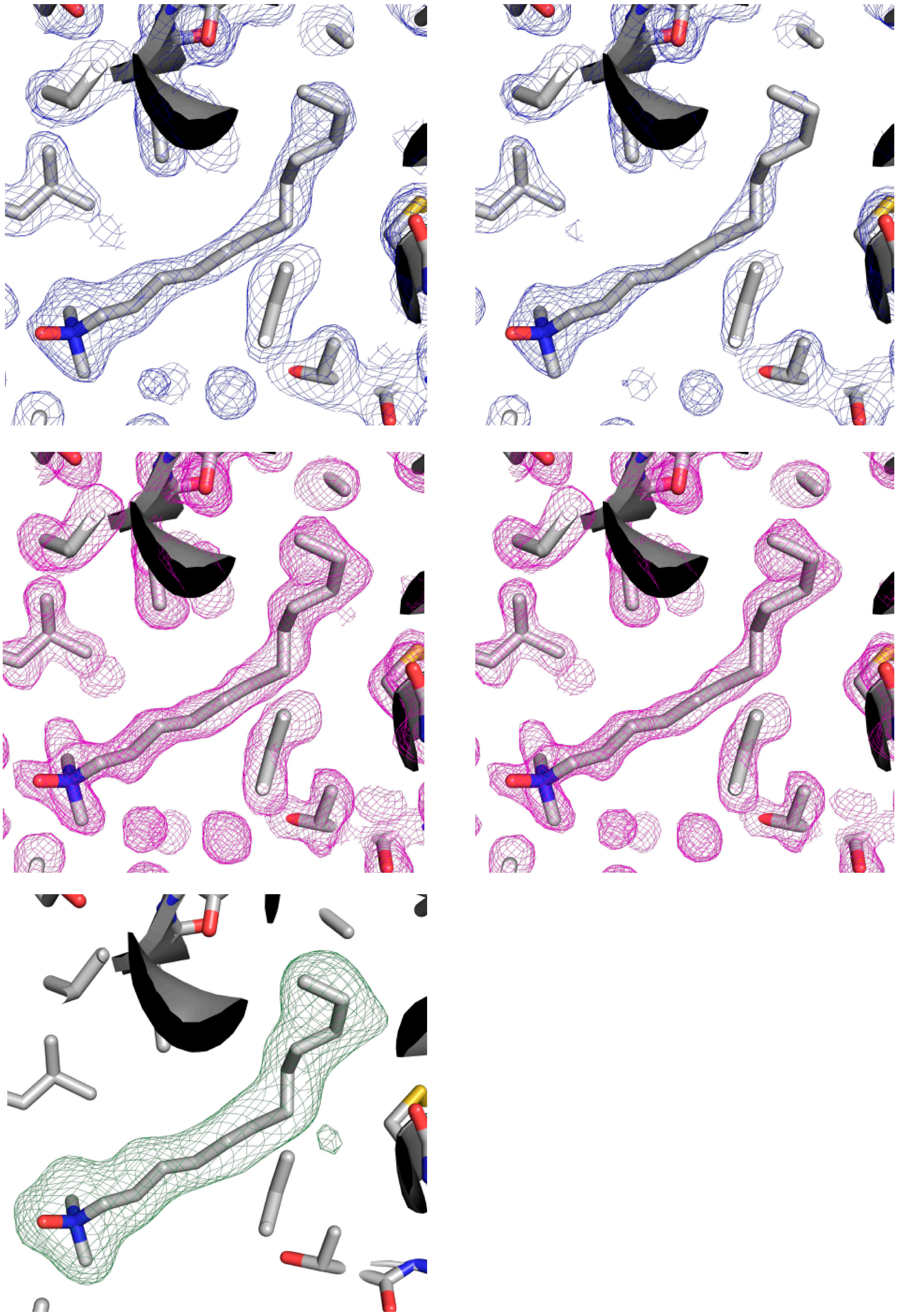

Figure S3. Electron density maps of PFL acyl-binding pocket. (a) Original 2mFobs-DFmodel (left, 1σ contour; right, 1.5σ contour). (b) Feature-enhanced map (left, 1σ contour; right, 1.5σ contour) [4]. (c) Polder map for ligand LDAO, contoured at +/-3σ [5].

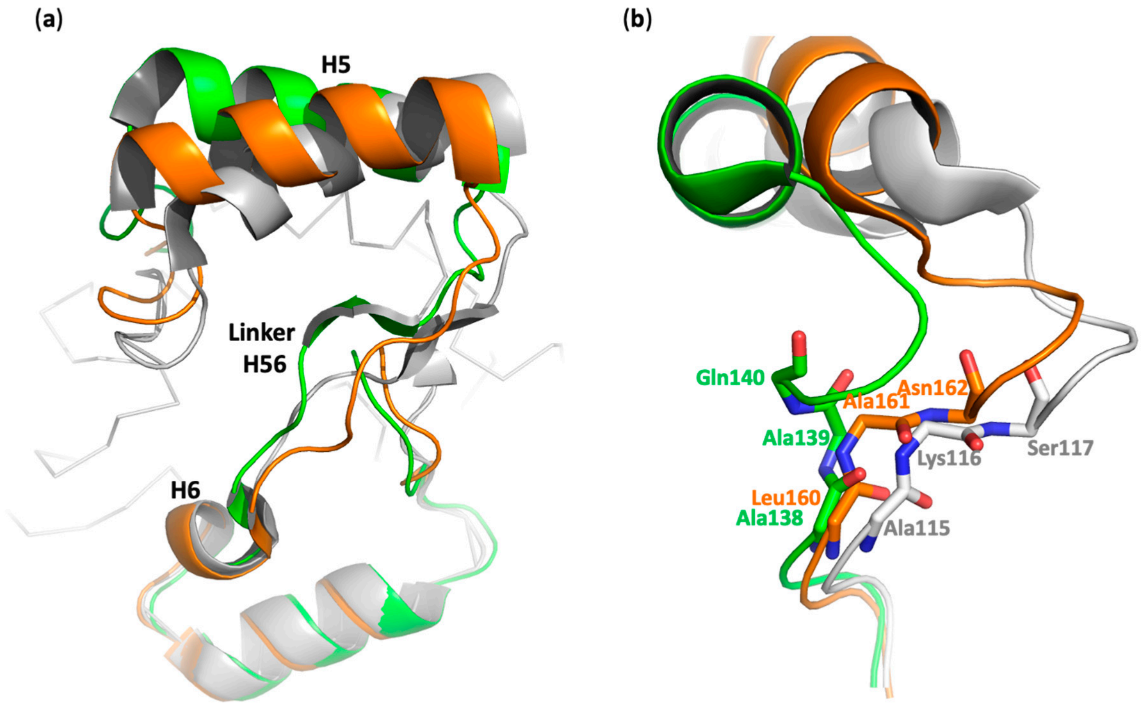

Figure S4. (**a**) Overlay of the structures of PFL, hMGL (pdb code 3jw8, chain A) and mtMGL (pdb code 6eic, chain B), which shows a conservation of the cap of these lipases. PFL is represented as ribbon, with its cap as cartoon. For hMGL and mtMGL, only their cap is represented. The position of H5, H6, and linker H56 is indicated (PFL, gray; hMGL, orange; mtMGL, green). (**b**) Comparison of the N-terminal hinge of H5 helix in PFL (gray), mtMGL (pdb code 6eic, chain B) and semi-open hMGL (pdb code 3jw8, chain A, orange). The psi angles of mtMGL residues 138-140, represented as sticks, differ compared to hMGL and PFL, yielding to a slightly different positioning of the H5 helix.

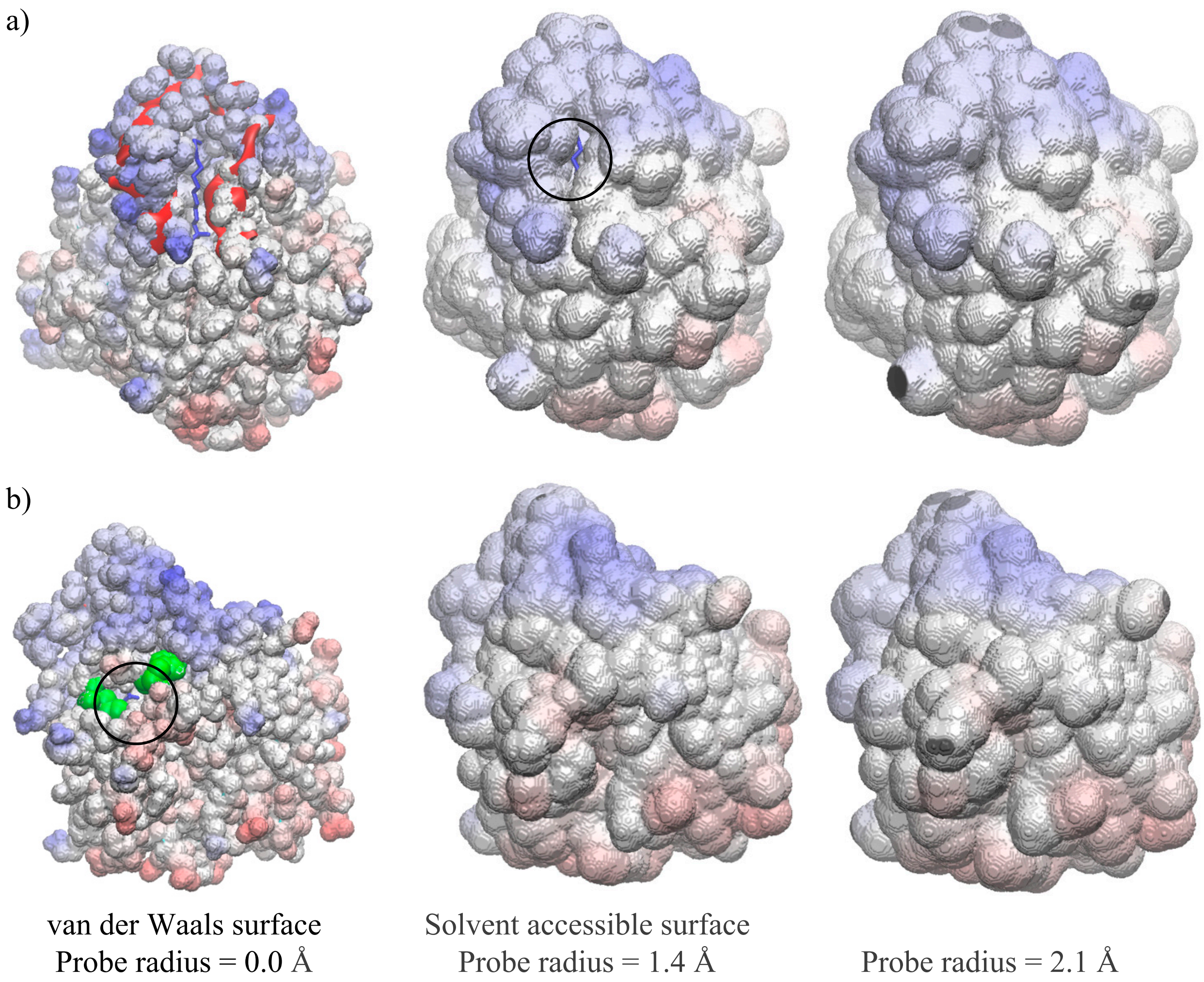

Figure S5A. Accessible surface of the crystal structure PFL chain A calculated using different probe radius values. (a) Front view, (b) side view. LDAO is shown with blue sticks. The surface is colored according to the Coulomb electrostatic potential calculated using the Gromos54a7 charges, from -0.5 (red) to 0.5 a.u. (blue). Leu144 and His166 are shown in green. The red curve and the circles help the reader to locate the lid and LDAO, respectively.

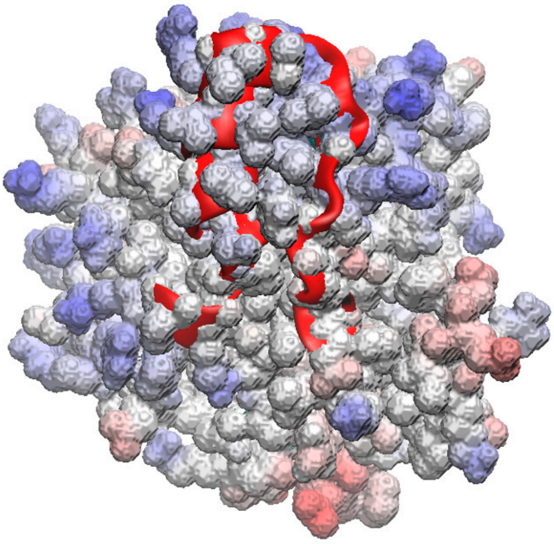

Figure S5B. Electrostatic potential distribution (from -0.5 to 0.5 a.u.), calculated using the Gromos54a7 charges, mapped onto the van der Waals surface of the PFL observed in the system PFL-G at t = 102.92 ns, as obtained from the MD trajectory at 300 K and 1 bar. The red curve helps the reader to locate the lid.

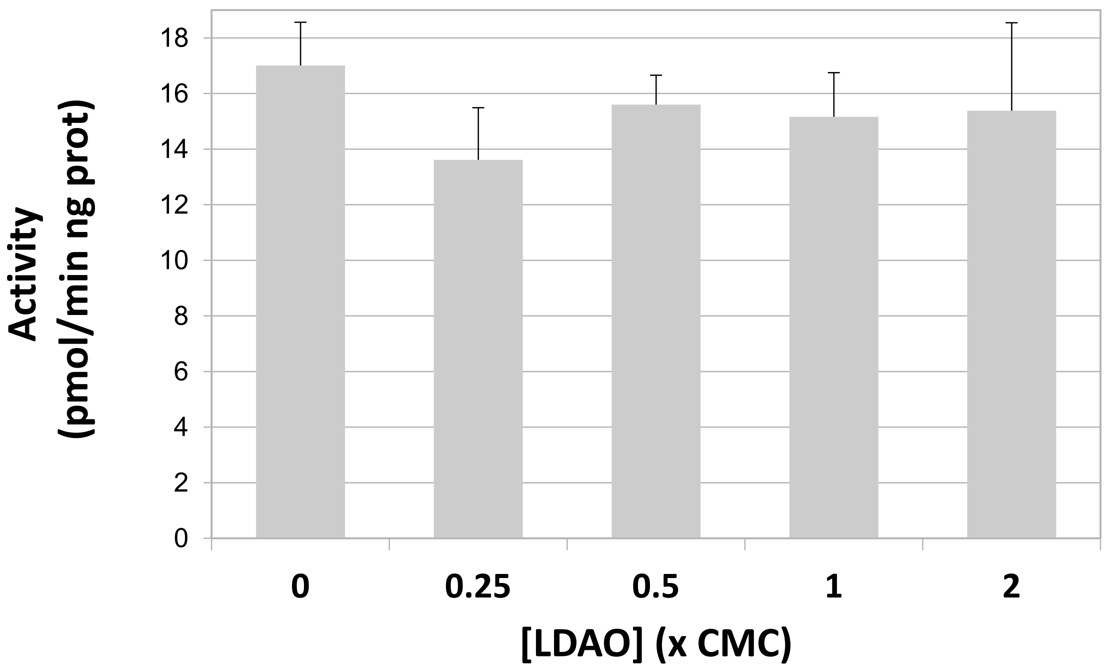

Figure S6. Measurement of PFL lipase activity in the presence of LDAO detergent. Hydrolase activity on p-nitrophenyl acetate was measured at 70°C in the presence of LDAO concentrations ranging from 0.25 to 2 times the critical micellar concentration (CMC). The amount of generated product was calculated after measuring the absorbance at 445 nm and blank subtraction. Values are the mean ± standard deviation of three experiments performed in duplicate.

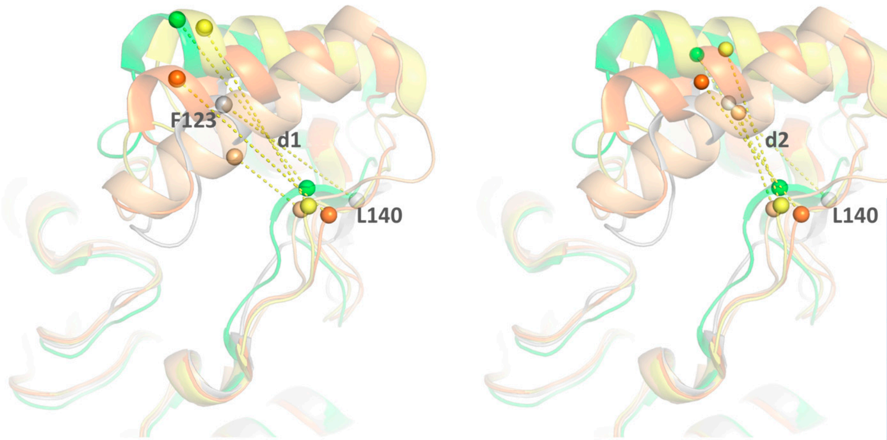

	d1	d2
**PFL**	14.9	11.9
**hMGL open**	16.4	13.3
**hMGL, semi-open**	16.6	13.4
**hMGL, closed**	6.8	8.5
**mtMGL**	17.0	12.9

Figure S7. Comparison of the opening state of PFL, hMGL (open, semi-open, and closed conformation), and mtMGL (open state). The distance between PFL Leu140 Cα, or the corresponding residue in homologous enzymes, and PFL Phe123 Cα (d1, left) or the centre of mass of residues 121-131(d2, right) in the H5 helix is indicated. Gray, PFL; yellow open MGL (pdb 4uuq, chain B); orange, semi-open MGL (pdb code 3jw8, chain A); light orange, closed hMGL (pdb code 3pe6); green, mtMGL (pdb code 6eic, chain B).

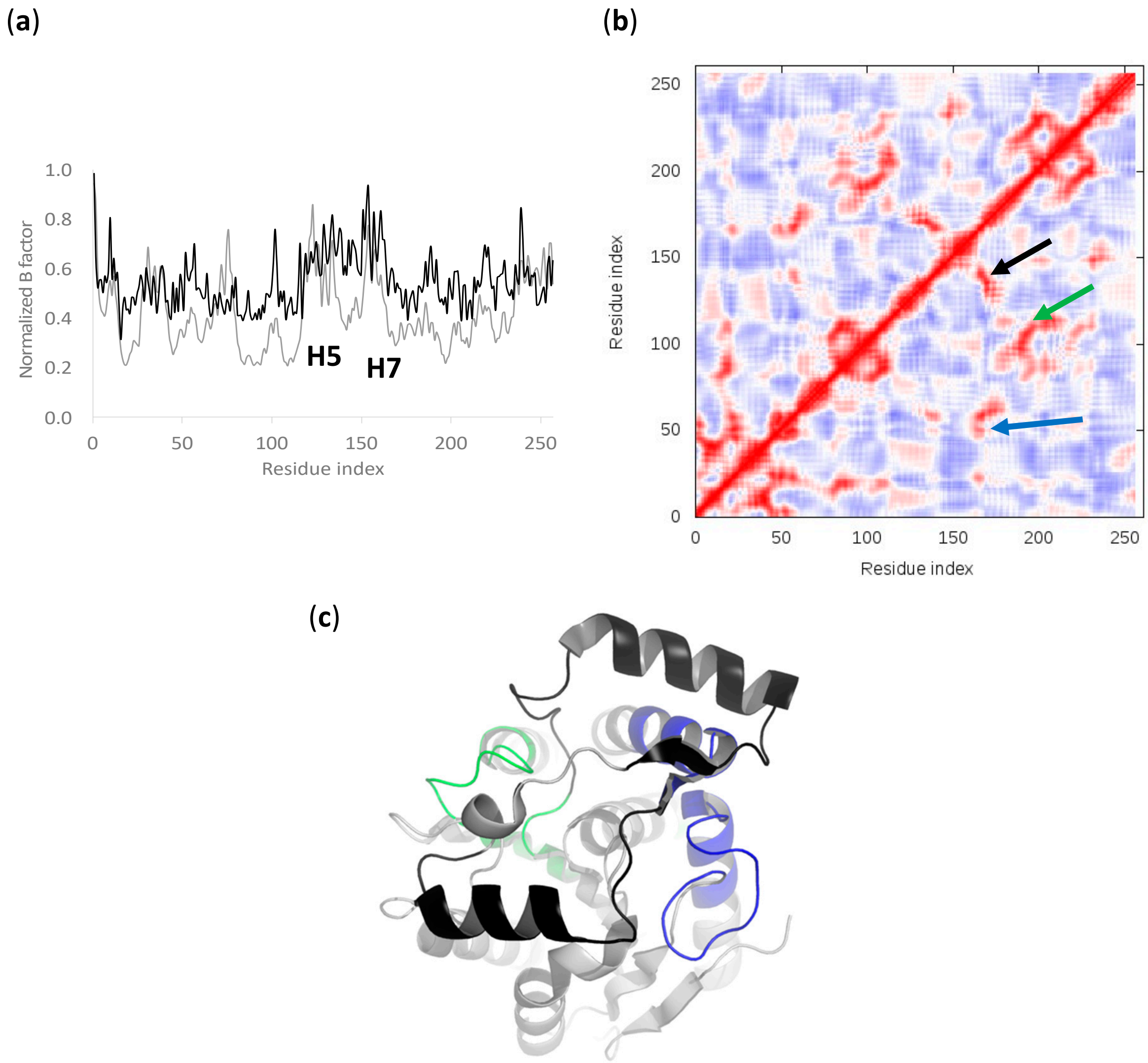

Figure S8. (**a**) iMODS (gray line) and experimental (black line) B factor profiles of the Cα atoms of the chain A of PFL. The two most mobile sequences correspond to the helices H5 and H7. (**b**) iMODS Cα covariance map (anti-correlated blue, uncorrelated white, correlated red). Arrows point to selected correlated motions**.** (**c**) 3D structure of the chain A of PFL colored according to the correlated structural elements identified by the corresponding arrows in (**b**).

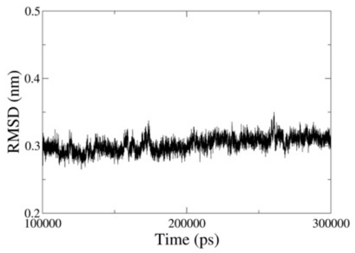

PFL

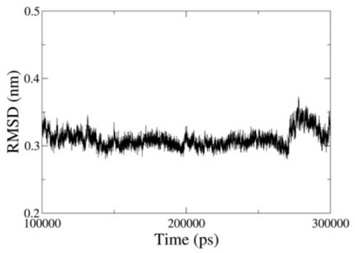

PFL-G

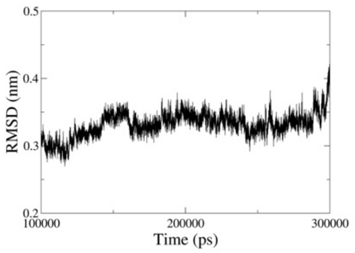

PFL-GL

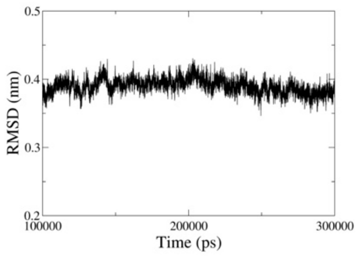

PFL-GM

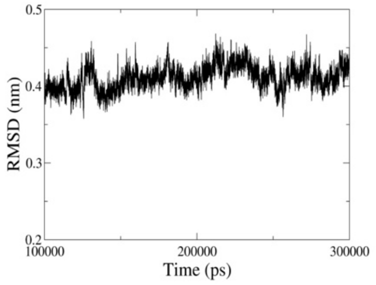

A (343 K)

Figure S9. RMSD profiles calculated for the PFL structure versus its initial conformation, as obtained from the last 200 ns of MD trajectories at 300 K or 343 K and 1 bar.

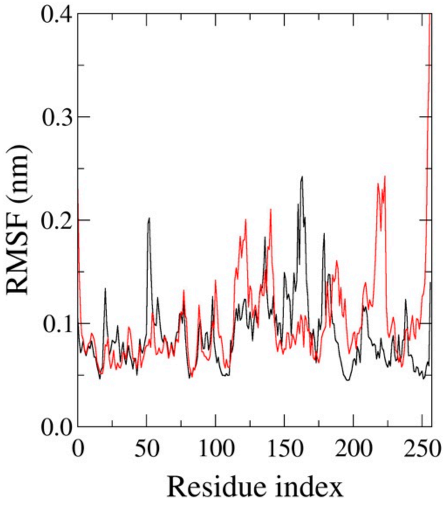

(a) PFL (black 300 K; red 343 K)

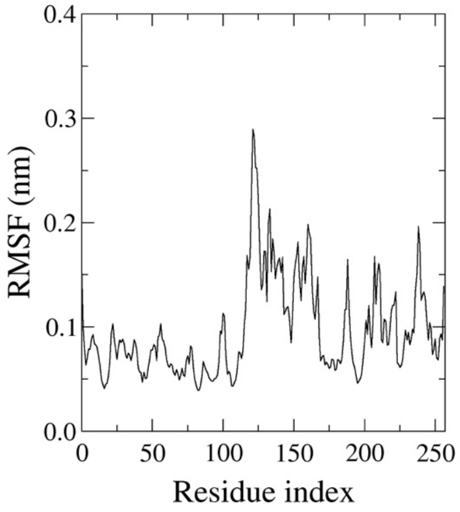

(b) PFL-G

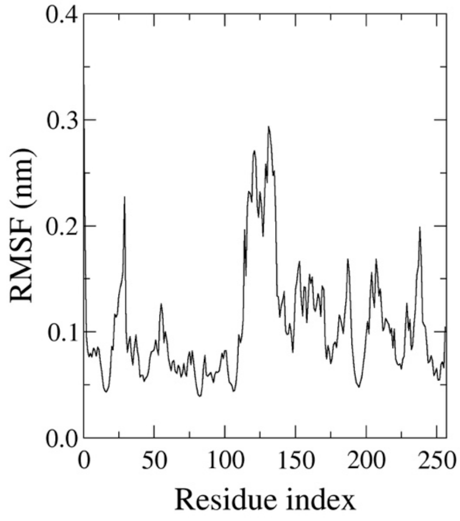

(c) PFL-GL

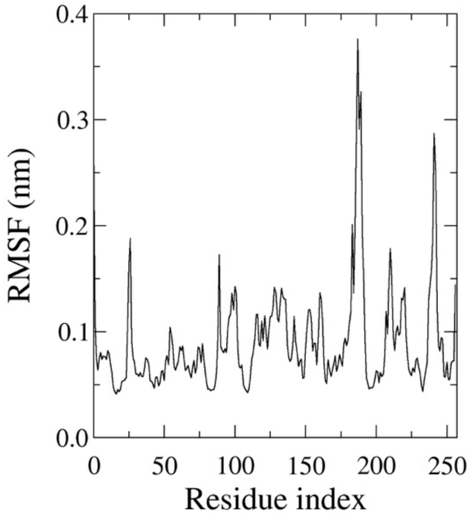

(d) PFL-GM

Figure S10. Cα RMSF profile as obtained from the last 200 ns of the MD trajectories at 300 K or 343 K and 1 bar.

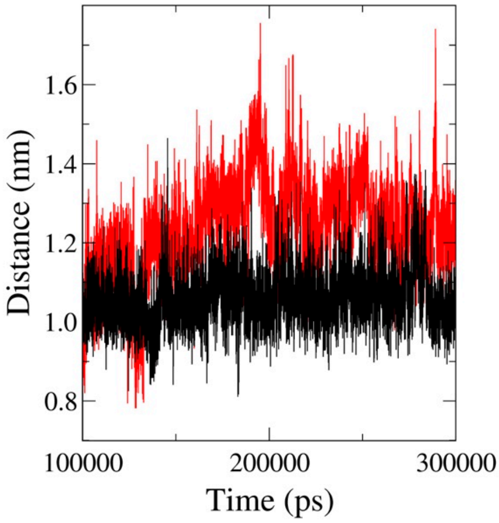

PFL
(300 K: 1.061 ± 0.071)
(343 K: 1.240 ± 0.117)

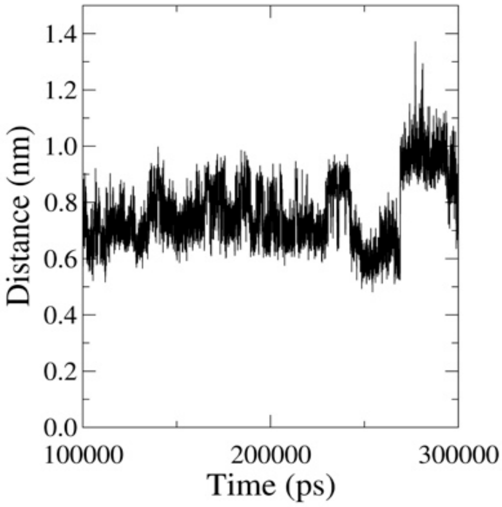

PFL-G
(0.764 ± 0.120)

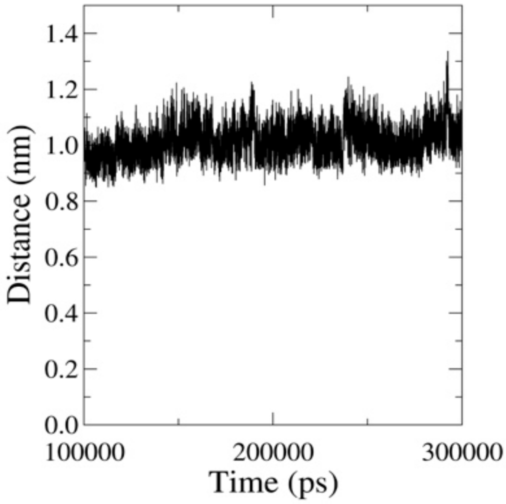

PFL-GL
(1.014 ± 0.061)

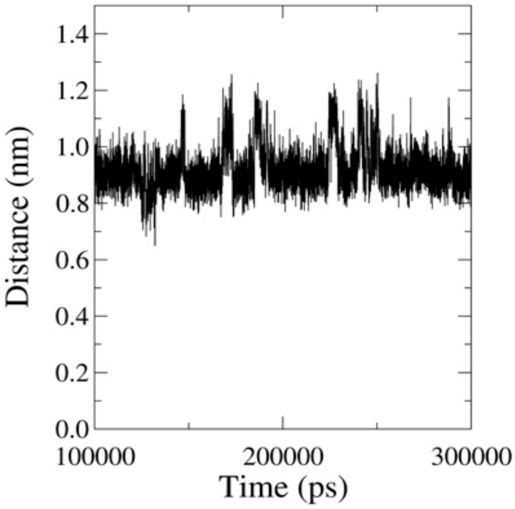

PFL-GM
(0.922 ± 0.076)

Figure S11A. Cα123-Cα140 distance profiles calculated for the PFL structure, as obtained from the last 200 ns of MD trajectories at 300 K or 343 K and 1 bar. The crystal structure value is 1.493 nm. Mean and standard deviation values (nm) are reported in parentheses.

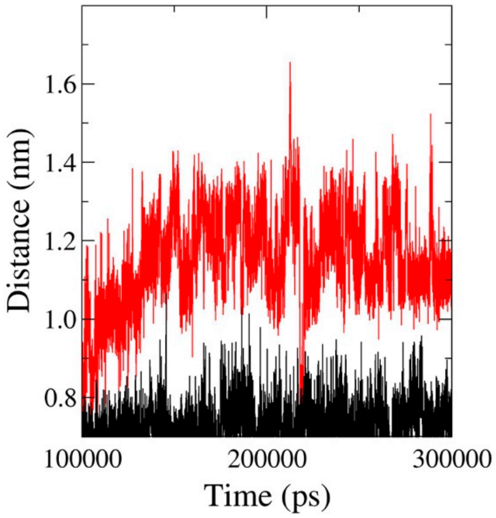

PFL
(300 K: 0.722 ± 0.063)
(343 K: 1.147 ± 0.126)

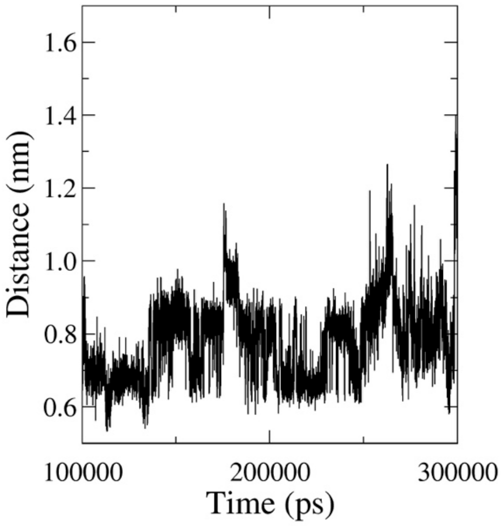

PFL-G
(0.779 ± 0.112)

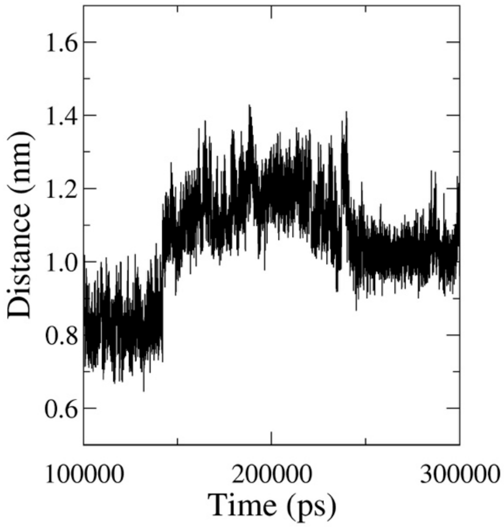

PFL-GL
(1.048 ± 0.144)

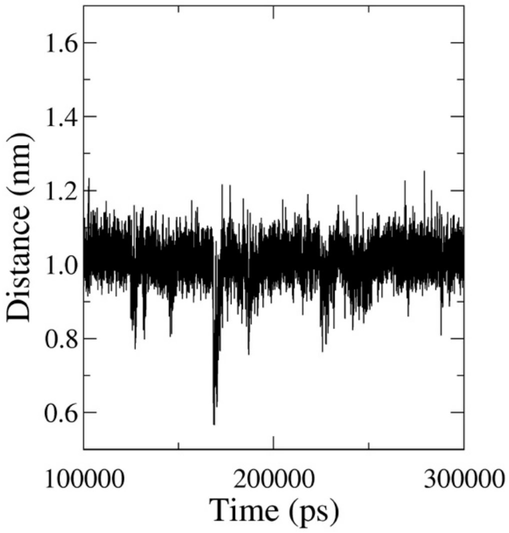

PFL-GM
(1.006 ± 0.061)

Figure S11B. Cα124-Cα142 distance profiles calculated for the PFL structure, as obtained from the last 200 ns of MD trajectories at 300 K or 343 K and 1 bar. The crystal structure value is 1.076 nm. Mean and standard deviation values (nm) are reported in parentheses.

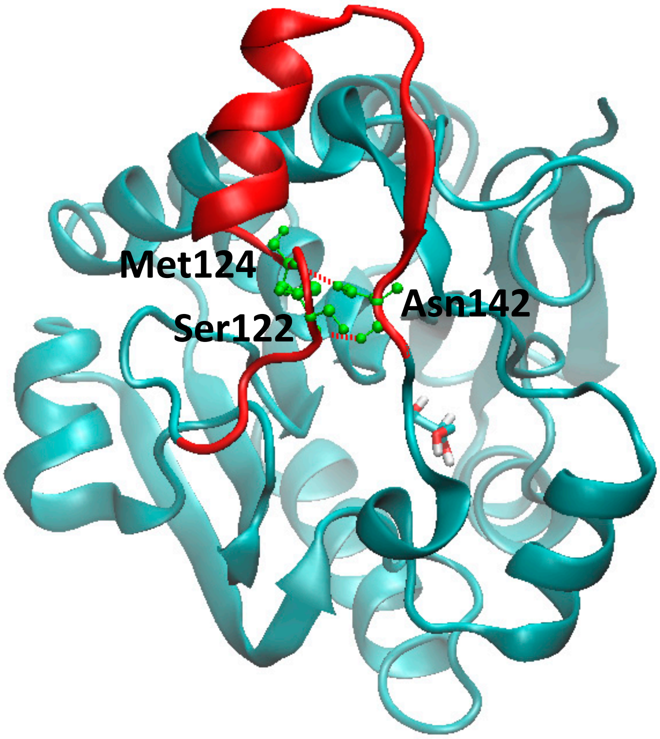

Figure S12A. Snapshot at t = 132.74 ns of the system PFL-G showing two hydrogen bonds formed by Asn142 with Ser122 and Met124, as obtained from a MD trajectory at 300 K and 1 bar. The enzyme lid is in red.

(a)

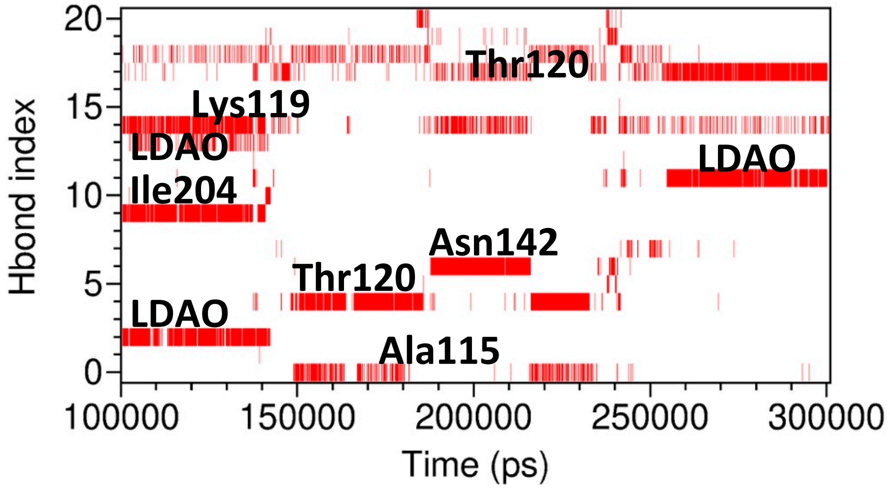

(b)

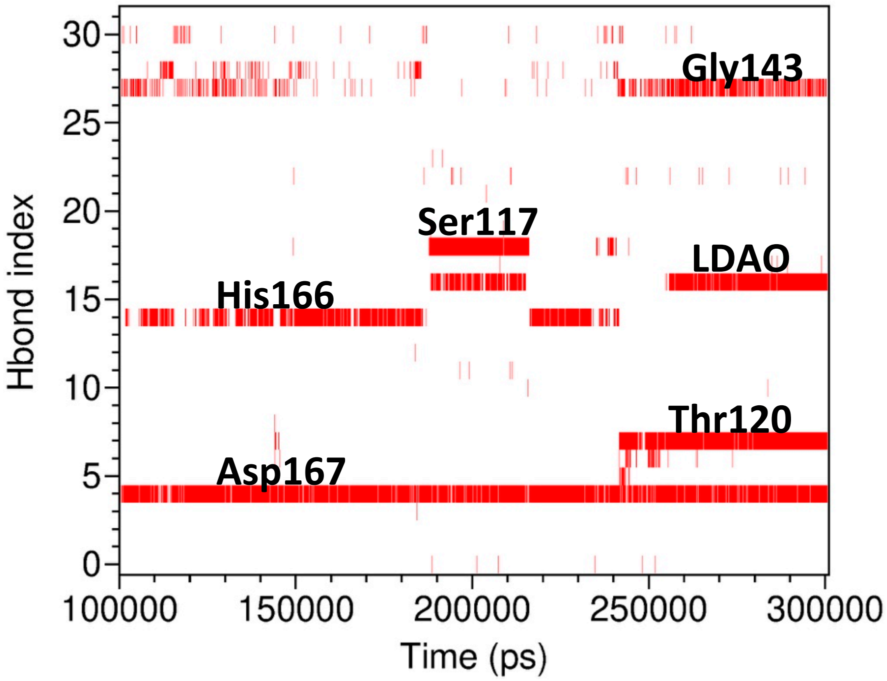

Figure S12B. Hydrogen bonds formed by (**a**) Ser117 and (**b**) Asn142 as obtained from the last 200 ns of the PFL-GL MD simulations at 300 K and 1 bar.

(**a**)

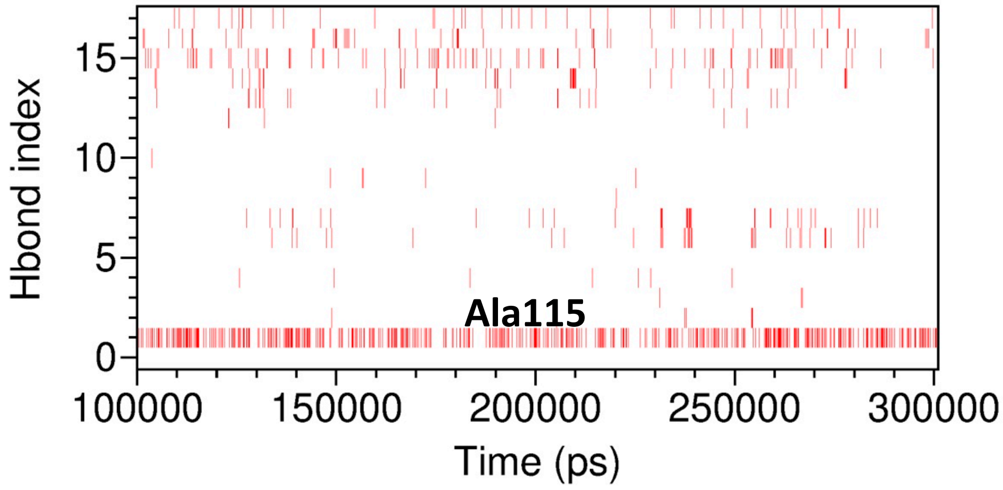

(**b**)

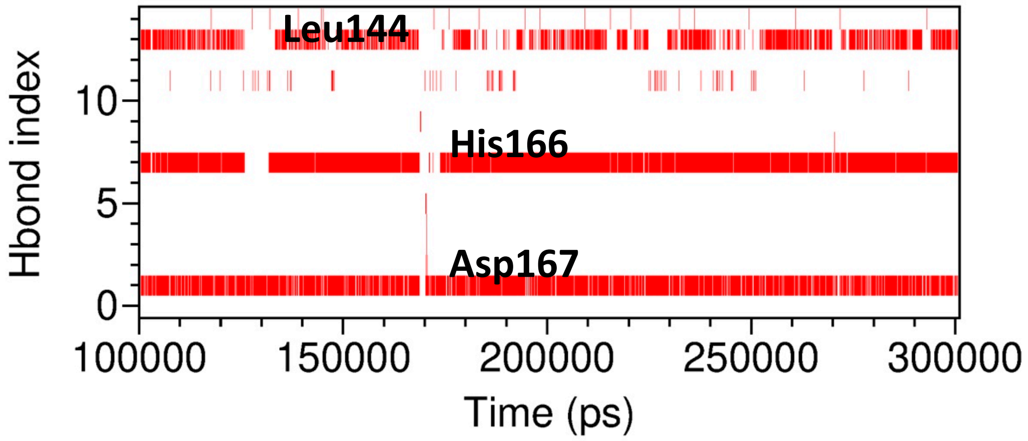

Figure S12C. Hydrogen bonds formed by (**a**) Ser117 and (**b**) Asn142 as obtained from the last 200 ns of the PFL-GM MD simulations at 300 K and 1 bar.

(a)

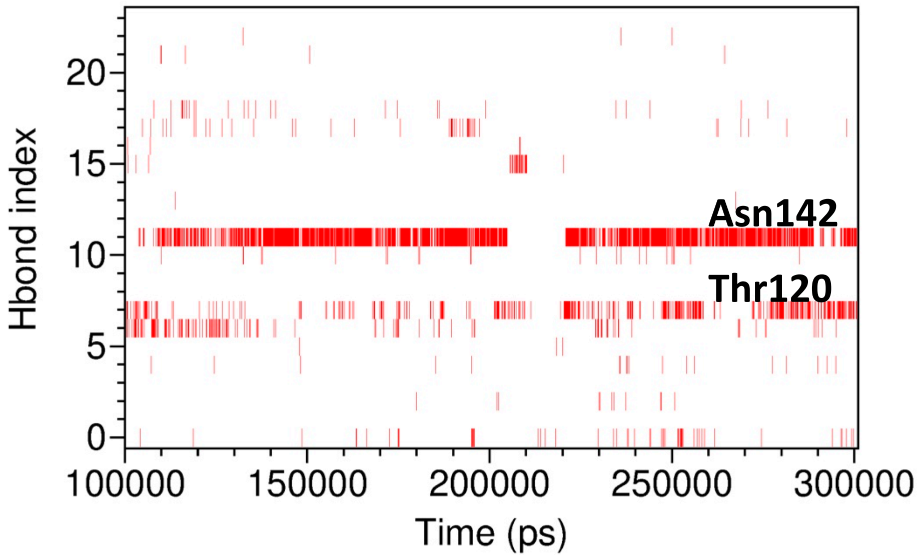

(b)

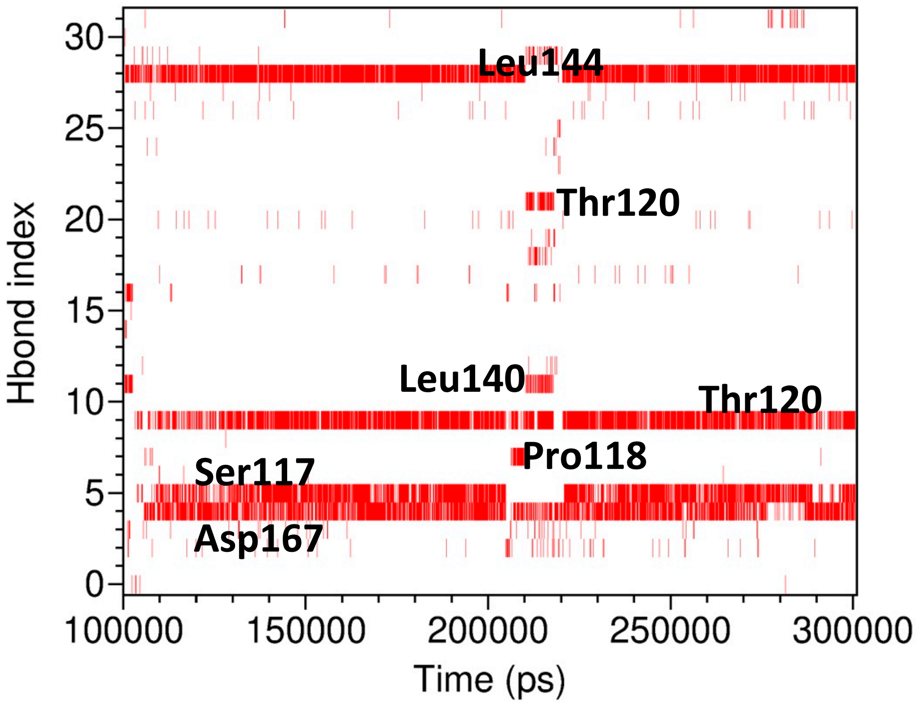

Figure S12D. Hydrogen bonds formed by (**a**) Ser117 and (**b**) Asn142 as obtained from the last 200 ns of the PFL-70 MD simulations at 343 K and 1 bar.

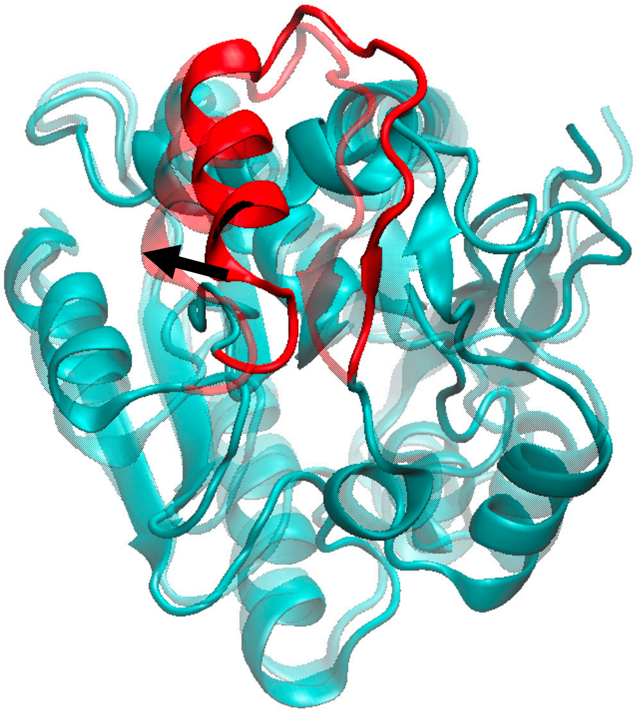

Figure S12E. Superimposition of two conformations of the system PFL-GL, at t = 200 ns (hydrogen bond between residues 117 and 204, opaque) and t = 300 ns (no hydrogen bond between residues 117 and 204, transparent), as obtained from the last 200 ns of the PFL-GL MD simulations at 300 K and 1 bar. The arrow illustrates the shift of the H5 helix. The enzyme lid is in red.

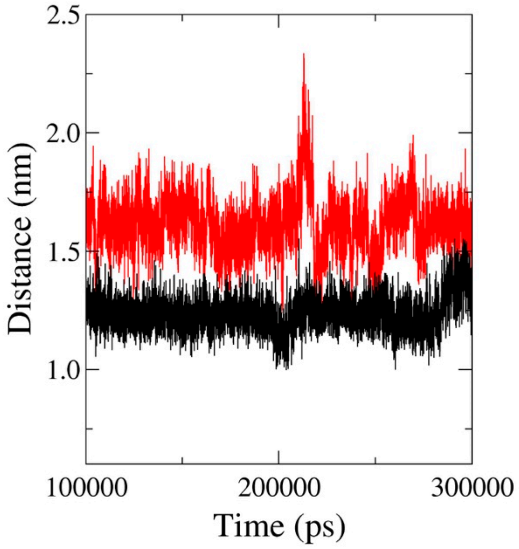

PFL
(300 K: 1.236 ± 0.074)
(343 K: 1.610 ± 0.115)

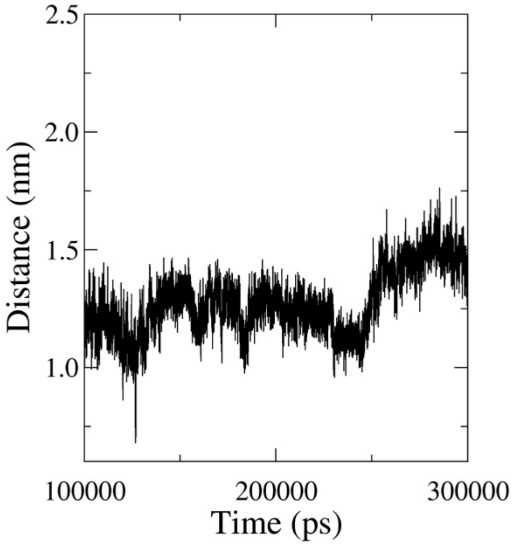

PFL-G
(1.275 ± 0.136)

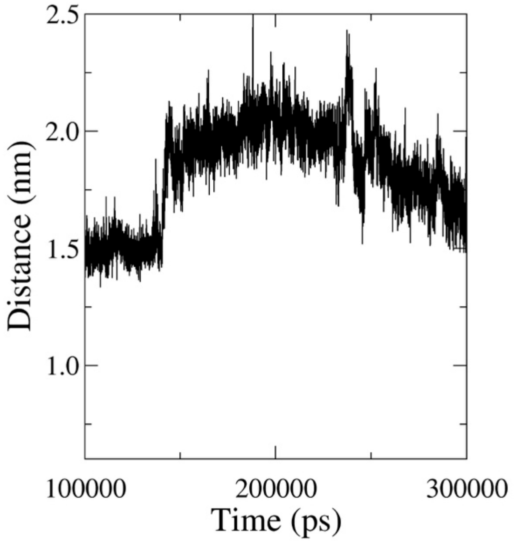

PFL-GL
(1.830 ± 0.212)

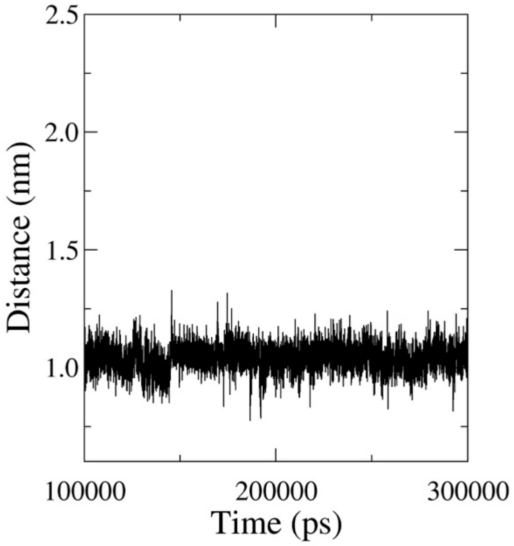

PFL-GM
(1.039 ± 0.052)

Figure S12F. Cα122-Cα204 distance profiles calculated for the PFL structure, as obtained from the last 200 ns of MD trajectories at 300 K or 343 K and 1 bar. The crystal structure value is 1.515 nm. Mean and standard deviation values (nm) are reported in parentheses.

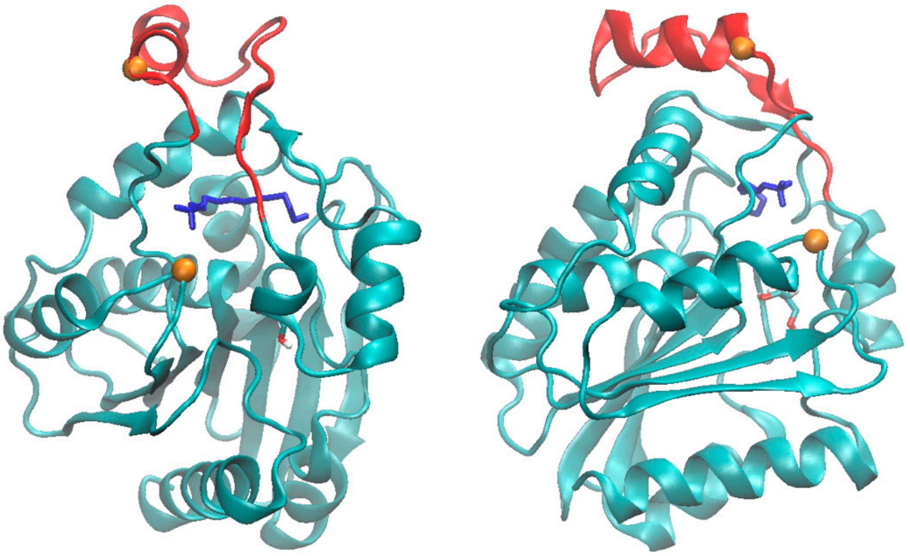

Front view Side view

Figure S12G. Snapshots at t = 188.14 ns of the system PFL-GL as obtained from a MD trajectory at 300 K and 1 bar. The enzyme lid is shown in red. The Cα atoms of residues 122 and 204 are in orange.

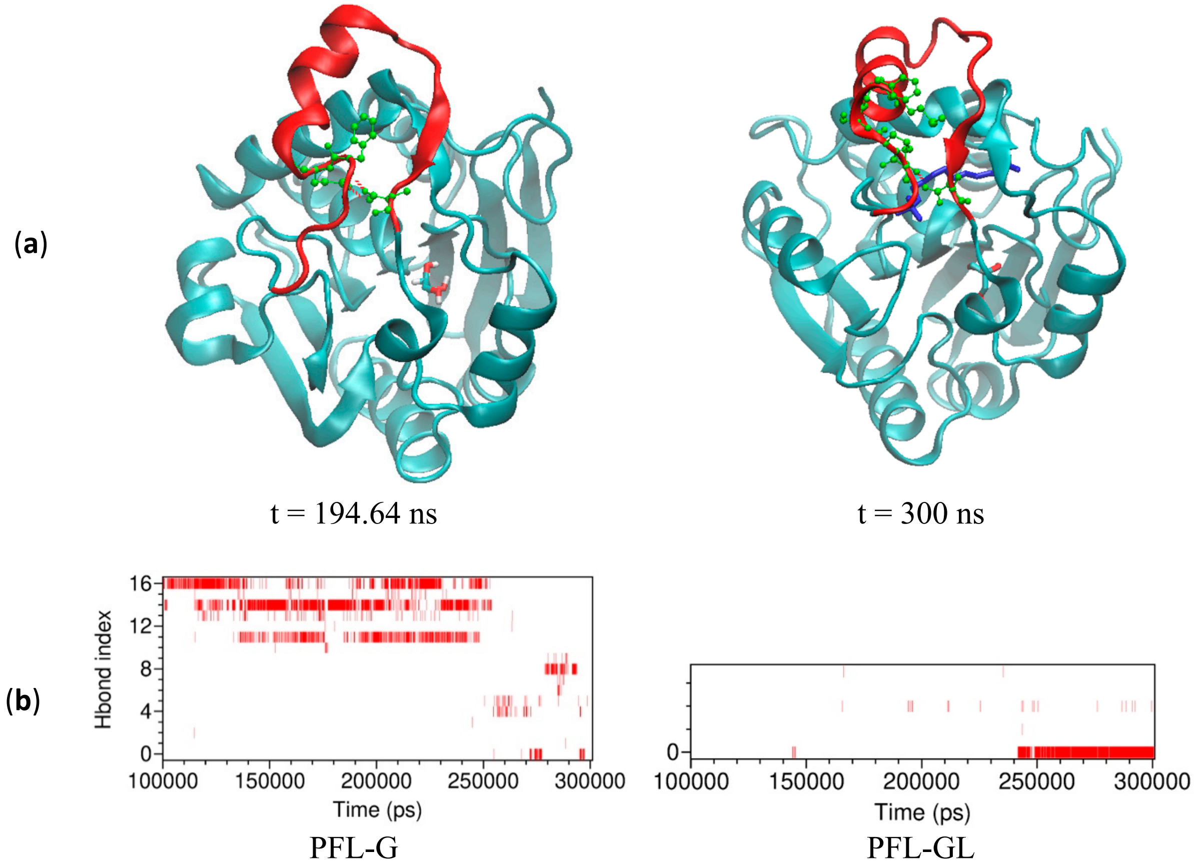

Figure S13. (**a**) Snapshots of the MD trajectories showing hydrogen bonds (red dashes) formed by Asn142 and facing residues (green). Residues 119 to 143 are in red. The enzyme lid is in red. (**b**) Hydrogen bond maps as calculated from the last 200 ns of MD trajectories at 300 K and 1 bar. The red bars indicate the presence of a hydrogen bond involving the residue Asn142.

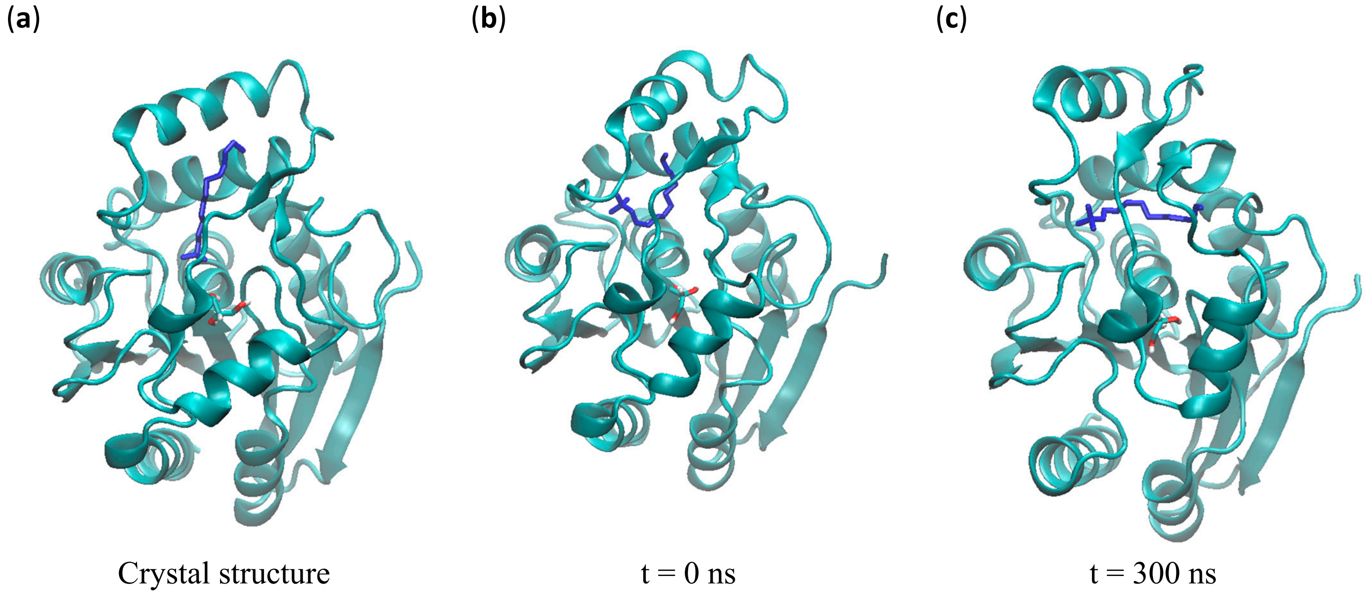

Figure S14. Chain A of PFL (cyan) with glycerol (colored sticks) and LDAO (dark blue) as obtained from (**a**) the crystallographic experiment, (**b**) at t = 0 ns, (**c**) at the end of the PFL-GL MD production stage.

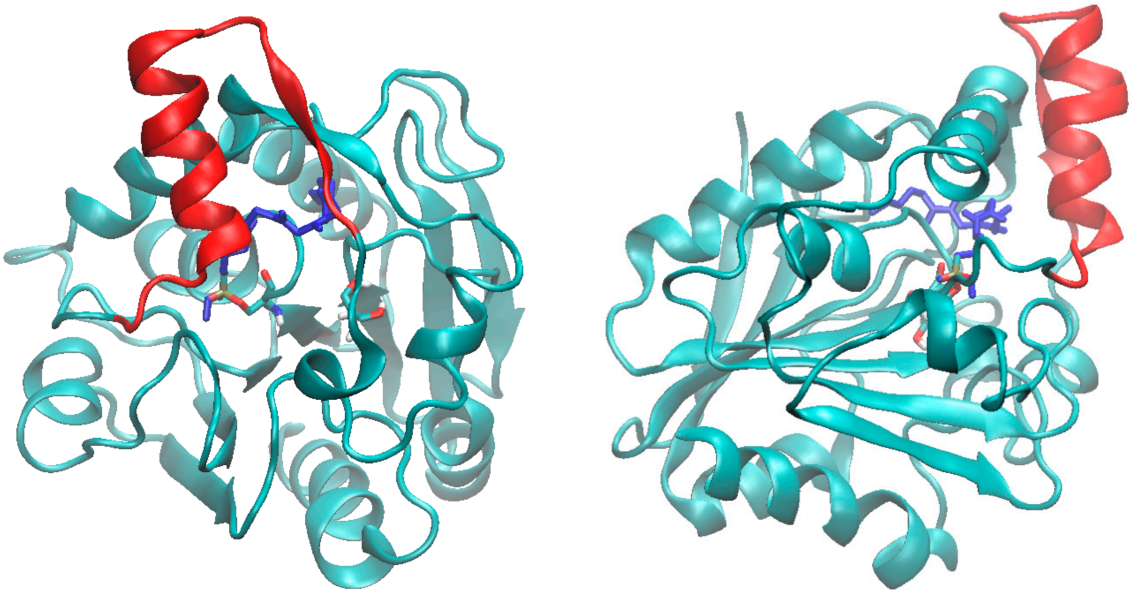

Front view   Side view

Figure S15. Snapshot at t = 130 ns of the system PFL-GM showing the folded conformation of the ligand (dark blue), as obtained from a MD trajectory at 300 K and 1 bar. The enzyme lid is shown in red.

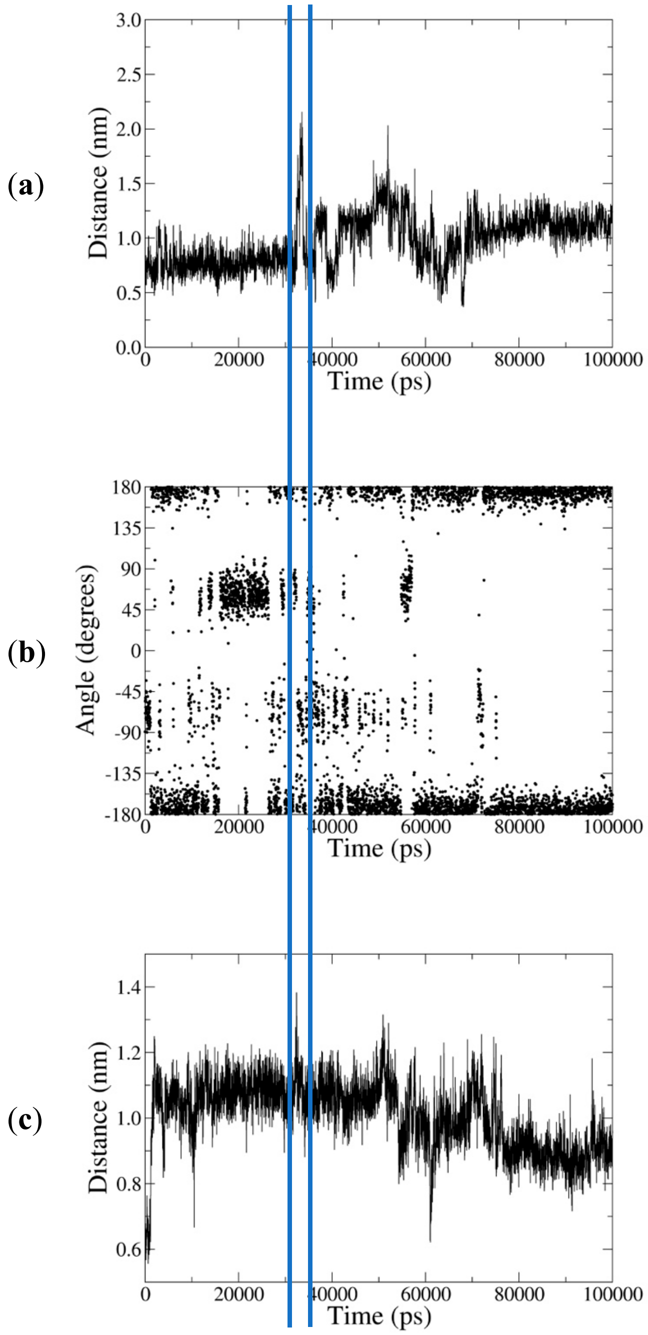

Figure S16. (**a**) MAFP distance profile P-C26, (**b**) dihedral N-Cα-Cβ-Cγ of Phe123 in PFL-GM, and (**c**) Cα123-Cα140 distance profiles calculated for the PFL structure in PFL-GM, as obtained from the last 100 ns of the MD equilibration stage at 300 K and 1 bar. The period of time corresponding to the crossing of MAFP through the enzyme surface is delimited by blue lines.

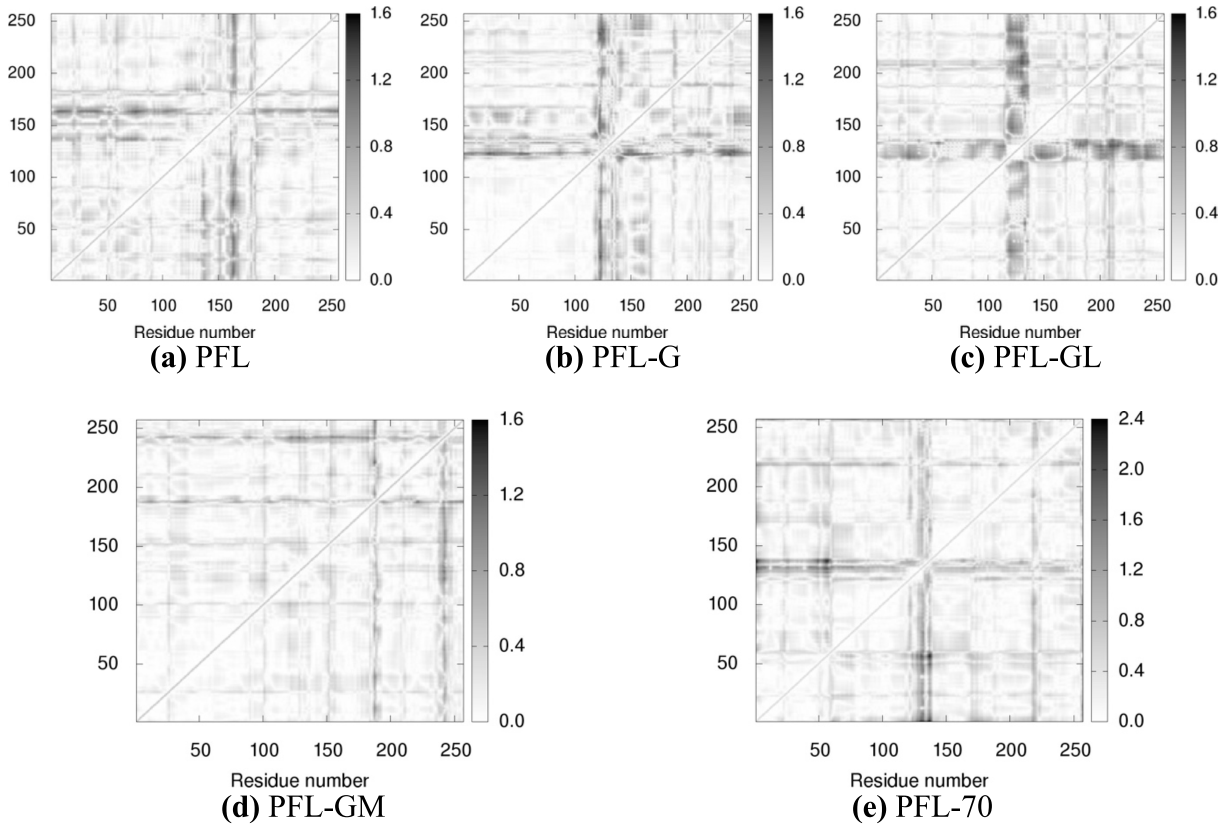

(a) PFL **(b)** PFL-G **(c)** PFL-GL

(d) PFL-GM **(e)** PFL-70

Figure S17. |κ.Δd| maps calculated using the first PC of the Cα covariance matrix, obtained from the last 200 ns of MD trajectories at 300 K or 343 K and 1 bar.

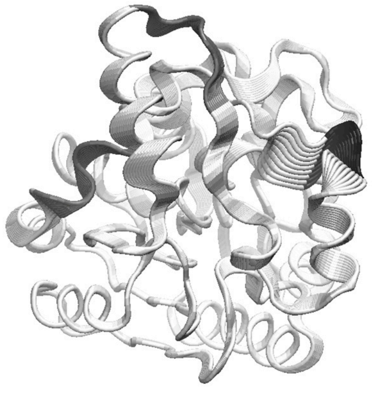

(a) PFL (white)
162-164 (black)
135-140 and 178-182 (gray)

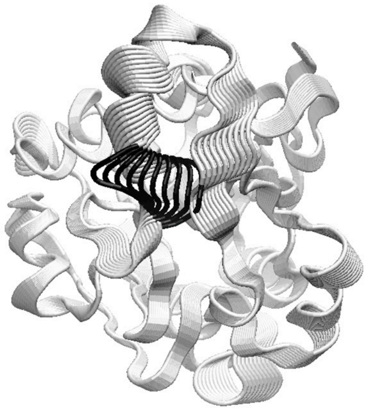

(b) PFL-G (white)
121-123 (black)

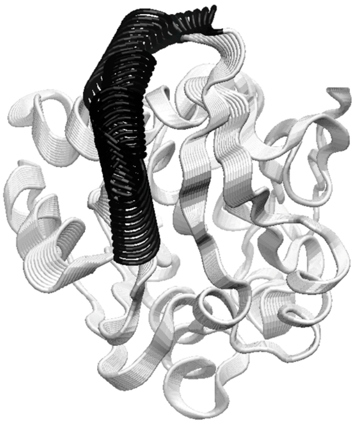

(c) PFL-GL (white)
117-135 (black)

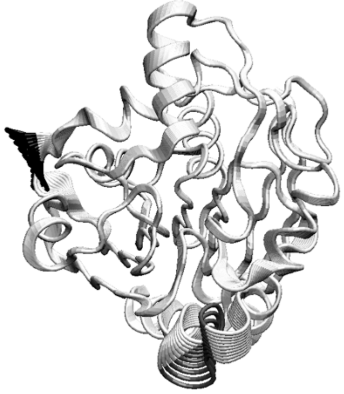

(d) PFL-GM (white)
187-189 (black)
241-243 (gray)

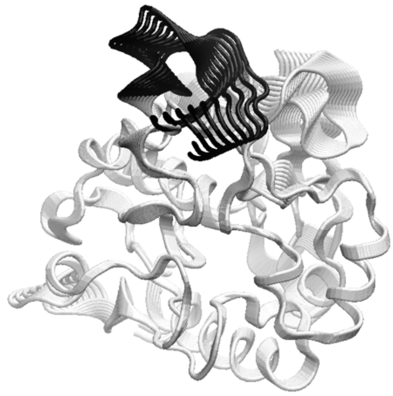

(e) PFL-70 (white)
127-139 (black)

Figure S18. PFL motion modelled using the first PC of the Cα covariance matrix, obtained from the last 200 ns of MD trajectories at 300 K or 343 K and 1 bar.

Table S1. Data collection and refinement statistics (Statistics for the highest-resolution shell are shown in parentheses).

	**PFL-6QE2**
Wavelength	0.98012
Resolution range	32.88 - 1.75 (1.813 - 1.75)
Space group	C 1 2 1
Unit cell	120.6 75.46 85.44 90 128.29 90
Total reflections	220730 (21657)
Unique reflections	60367 (5905)
Multiplicity	3.7 (3.7)
Completeness (%)	99.39 (98.15)
Mean I/sigma(I)	13.85 (2.14)
Wilson B-factor	28.06
R-merge	0.04658 (0.3796)
R-meas	0.05434 (0.4433)
R-pim	0.02771 (0.2266)
CC1/2	0.999 (0.931)
CC*	1 (0.982)
Reflections used in refinement	60366 (5886)
Reflections used for R-free	3018 (295)
R-work	0.1710 (0.2969)
R-free	0.1980 (0.3425)
CC(work)	0.967 (0.925)
CC(free)	0.961 (0.893)
RMS(bonds)	0.008
RMS(angles)	1.17
Ramachandran favored (%)	97.84
Ramachandran outliers (%)	0.00
Rotamer outliers (%)	0.00

Table S2. Helix content of chain A of protein PFL as obtained from the crystal structure.

Helix code	First residue		Last residue		Helix length
H1	HIS	25	ARG	28	4
H2	TYR	29	ALA	39	11
H3	SER	62	GLY	78	17
H4	SER	87	ARG	100	14
H5	PRO	121	THR	135	15
H6	ASP	145	LEU	149	5
H7	ASN	152	ASP	162	11
H8	GLY	171	GLU	187	17
H9	ALA	188	ILE	191	4
H10	PRO	208	LEU	218	11
H11	TRP	241	SER	257	17

SM1. Technical details regarding the ligand building

To generate the Gromos54a7 FF parameters of the LDAO ligand, the ligand structure was submitted to the ATB server [6]. Topology files describing the ligand and the glycerol molecules at the united-atom level were directly retrieved from the ATB server, with the MolID 13206 and 30887, respectively.

To generate the enzyme-MAFP covalently bound complex, the PDB structure 1mt5 was aligned with chain A of PFL using Pymol (Pymol, version 1.8)[7]. It allowed to retrieve an orientation of the methyl arachidonyl fluorophosphonate ligand in adequation with the pocket of the chain A crystal structure. From that configuration, the residue Ser87 was linked to the ligand to generate the moiety reported in Figure SM1_1. The Serine N and C terminal groups were capped with electrically neutral $CH_3(C{=}O)$- (ACE) and -$N(CH_3)_2$ (NME) groups.

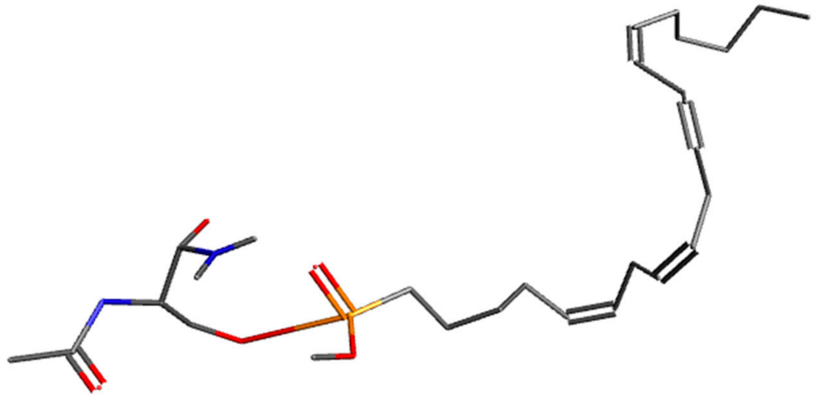

Figure SM1_1. Serine-MAFP $C_{28}H_{57}N_2O_5P$ conformation submitted to the ATB server [6]. H atoms are not shown for clarity.

The moiety topology was retrieved at the united-atom level (ATB MolID 368680) and added to (or replacing some of) the Ser87 topology generated by the Gromacs5.1.4 building tools, while preserving an electric charge of zero (Table SM1_1). An in-house program was written for the renumbering of the ligand atoms and the generation of the additional 1-4 interactions.

Table SM1_1. List of the ligand MAFP point charges obtained using the ATB server with the united-atom FF Gromos54a7.

Name	Residue	Label	Charge	Mass
N	SER	N	-0.31	14.0067
H	SER	H	0.31	1.008
CH1	SER	CA	-0.03	13.019
CH2	SER	CB	0.223	14.027
OA	SER	OG	-0.75	15.9994
P	MAFP	P1	2.699	30.9738
C	SER	C	0.45	12.011
O	SER	O	-0.45	15.9994
OM	MAFP	O4	-1.132	15.9994
OAlc	MAFP	O5	-0.752	15.9994
CH3	MAFP	C6	0.24	15.035
CH2	MAFP	C7	-0.576	14.027
CH2	MAFP	C8	0.055	14.027
CH2	MAFP	C9	0.024	14.027
CH2	MAFP	C10	0.04	14.027
C	MAFP	C11	-0.174	12.011
HC	MAFP	H11	0.124	1.008
C	MAFP	C12	-0.161	12.011
HC	MAFP	H10	0.125	1.008
CH2	MAFP	C13	0.083	14.027
C	MAFP	C14	-0.166	12.011
HC	MAFP	H9	0.124	1.008
C	MAFP	C15	-0.165	12.011
HC	MAFP	H8	0.125	1.008
CH2	MAFP	C16	0.081	14.027
C	MAFP	C17	-0.169	12.011
HC	MAFP	H7	0.125	1.008
C	MAFP	C18	-0.163	12.011
HC	MAFP	H6	0.125	1.008
CH2	MAFP	C19	0.083	14.027
C	MAFP	C20	-0.169	12.011
HC	MAFP	H5	0.123	1.008
C	MAFP	C21	-0.166	12.011
HC	MAFP	H4	0.123	1.008
CH2	MAFP	C22	0.041	14.027
CH2	MAFP	C23	0.008	14.027
CH2	MAFP	C24	0.001	14.027
CH2	MAFP	C25	-0.003	14.027
CH3	MAFP	C26	0.004	15.035

SM2. Description of the Molecular Dynamics (MD) calculations

MD trajectories of the solvated systems were run using the Gromacs5.1.4 program package [8] with the Gromos54a7 force field [9] under particle mesh Ewald periodic boundary conditions and a Coulomb cut-off distance of 1.2 nm. The Newton equations of motion were numerically integrated using a leap-frog integrator. The van der Waals cut-off distance was set equal to 1.2 nm. Long-range dispersion corrections to energy and pressure were applied. The systems were optimized using a steepest descent algorithm with an initial step size of 0.10 nm.

The whole systems were again optimized, using a steepest descent algorithm with an initial step size of 0.10 nm, to eliminate large forces and then heated to 50 K through a 10 ps canonical (NVT) MD, with a time step of 2 fs and LINCS constraints acting on bonds involving H atoms. The trajectory was followed by two successive 20 ps heating stages, at 150 K and at the final temperature of 300 K (or 343 K), under the same conditions. Next, each system was equilibrated during 50 ps in the NPT ensemble, at P = 1 bar, to relax the solvent molecules, and for a further 60 ns MD equilibration run. The 'V-Rescale' and 'Parrinello-Rahman' algorithms were selected to constrain T and P, respectively. A final production run of 300 ns (150 x 10^6 steps) was performed for the evaluation of the structural, energetics, and dynamical properties of each system. Trajectory data were saved every 20 ps. It was further decided to consider the last 200 ns of the simulations only to avoid the largest fluctuations of the systems. The various stages of the simulations are reported in Table SM2_1.

Table SM2_1. MD simulation conditions and stages.

Geometry optimization		
	Energy tolerance	1 kJ.mol^{-1}.nm^{-1}
	Max. no. of iterations	5000
Molecular Dynamics - Common parameters		
	Periodic boundary conditions	Cubic box
	Time step	2 fs
	Long-range electrostatics	Particle Mesh Ewald
	van der Waals cut-off	1.2 nm
	Integrator	Leap-frog
	Thermostat	V-rescale
	Barostat	Isotropic Parinello-Rahman
	Constraints	LINCS applied to chemical bonds involving H atoms
Molecular Dynamics - Equilibration stage 1		
	Temperature	50 K
	No. of iterations	5000
	Statistical ensemble	NVT
Molecular Dynamics - Equilibration stage 2		
	Temperature	150 K
	No. of iterations	10000
	Statistical ensemble	NVT
Molecular Dynamics - Equilibration stage 3		
	Temperature	300 K (or 343 K)
	No. of iterations	10000
	Statistical ensemble	NVT
Molecular Dynamics - Equilibration stage 4		
	Temperature	300 K (or 343 K)

	Pressure	1 bar
	No. of iterations	25000
	Statistical ensemble	NPT
Molecular Dynamics - Equilibration stage 5		
	Temperature	300 K (or 343 K)
	Pressure	1 bar
	No. of iterations	3 x 10 10^6
	Statistical ensemble	NPT
Molecular Dynamics - Production stage [a]		
	Temperature	300 K (or 343 K)
	Pressure	1 bar
	No. of iterations	150 10^6
	Statistical ensemble	NPT
	Dumping interval	20 ps

[a] The last 200 ns only are considered for the statistical analyses.

References

1. Madeira, F.; Park, Y.M.; Lee, J.; Buso, N.; Gur, T.; Madhusoodanan, N.; Basutkar, P.; Tivey, A.R.N.; Potter, S.C.; Finn, R.D.; et al. The EMBL-EBI Search and Sequence Analysis Tools APIs in 2019. *Nucleic Acids Res.* **2019**, *47*, W636–W641, doi:10.1093/nar/gkz268.
2. Riegler-Berket, L.; Leitmeier, A.; Aschauer, P.; Dreveny, I.; Oberer, M. Identification of Lipases with Activity towards Monoacylglycerol by Criterion of Conserved Cap Architectures. *Biochim. Biophys. Acta Mol. Cell Biol. Lipids* **2018**, *1863*, 679–687, doi:10.1016/j.bbalip.2018.03.009.
3. Laskowski, R.A.; Jabłońska, J.; Pravda, L.; Vařeková, R.S.; Thornton, J.M. PDBsum: Structural Summaries of PDB Entries. *Protein Sci. Publ. Protein Soc.* **2018**, *27*, 129–134, doi:10.1002/pro.3289.
4. Afonine, P.V.; Moriarty, N.W.; Mustyakimov, M.; Sobolev, O.V.; Terwilliger, T.C.; Turk, D.; Urzhumtsev, A.; Adams, P.D. FEM: Feature-Enhanced Map. *Acta Crystallogr. D Biol. Crystallogr.* **2015**, *71*, 646–666, doi:10.1107/S1399004714028132.
5. Liebschner, D.; Afonine, P.V.; Moriarty, N.W.; Poon, B.K.; Sobolev, O.V.; Terwilliger, T.C.; Adams, P.D. Polder Maps: Improving OMIT Maps by Excluding Bulk Solvent. *Acta Crystallogr. Sect. Struct. Biol.* **2017**, *73*, 148–157, doi:10.1107/S2059798316018210.
6. Malde, A.K.; Zuo, L.; Breeze, M.; Stroet, M.; Poger, D.; Nair, P.C.; Oostenbrink, C.; Mark, A.E. An Automated Force Field Topology Builder (ATB) and Repository: Version 1.0. *J. Chem. Theory Comput.* **2011**, *7*, 4026–4037, doi:10.1021/ct200196m.
7. *The PyMOL Molecular Graphics System, Version 1.8 Schrödinger, LLC.*;
8. Abraham, M.J.; Murtola, T.; Schulz, R.; Páll, S.; Smith, J.C.; Hess, B.; Lindahl, E. GROMACS: High Performance Molecular Simulations through Multi-Level Parallelism from Laptops to Supercomputers. *SoftwareX* **2015**, *1–2*, 19–25, doi:10.1016/j.softx.2015.06.001.
9. Schmid, N.; Eichenberger, A.P.; Choutko, A.; Riniker, S.; Winger, M.; Mark, A.E.; van Gunsteren, W.F. Definition and Testing of the GROMOS Force-Field Versions 54A7 and 54B7. *Eur. Biophys. J. EBJ* **2011**, *40*, 843–856, doi:10.1007/s00249-011-0700-9.
